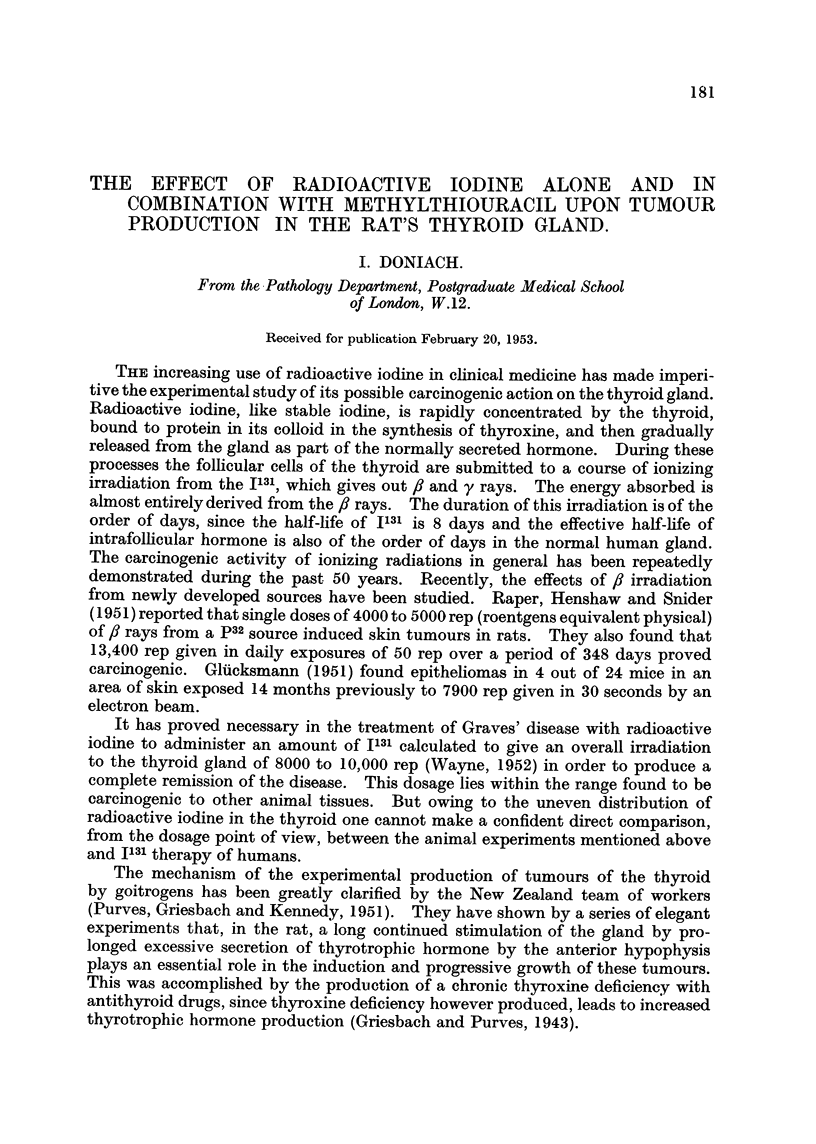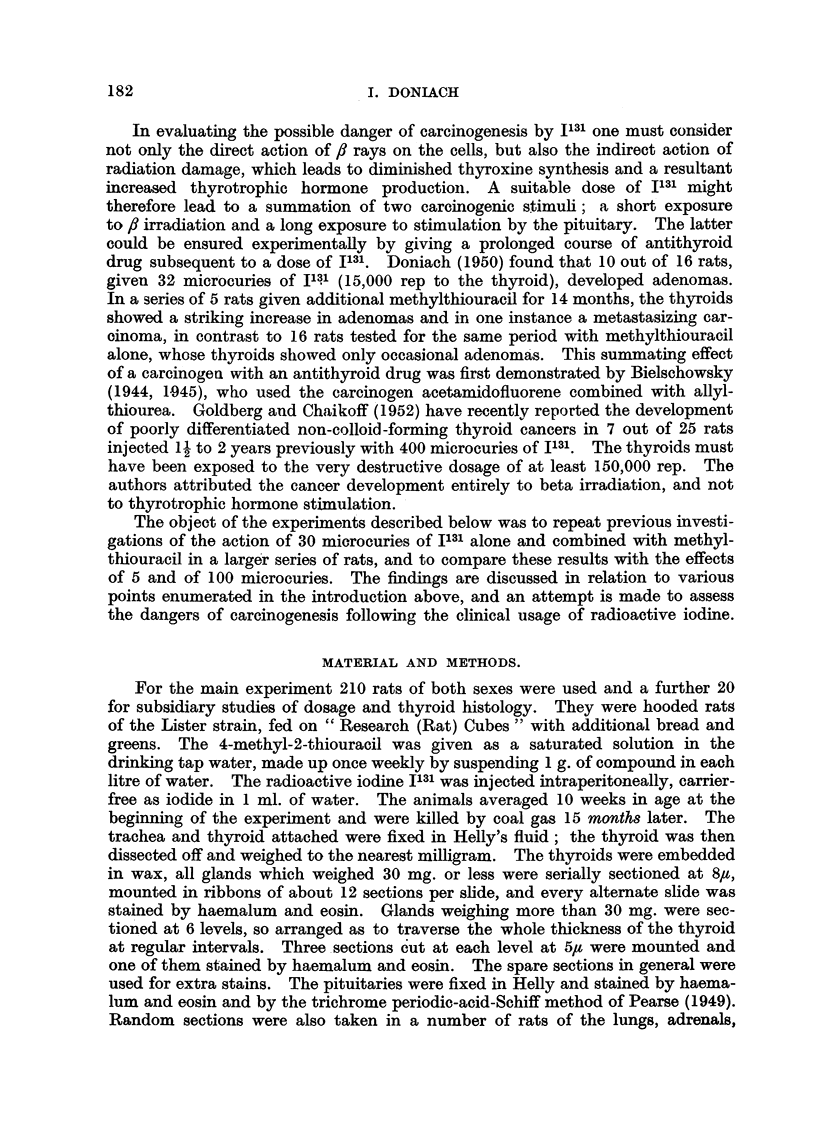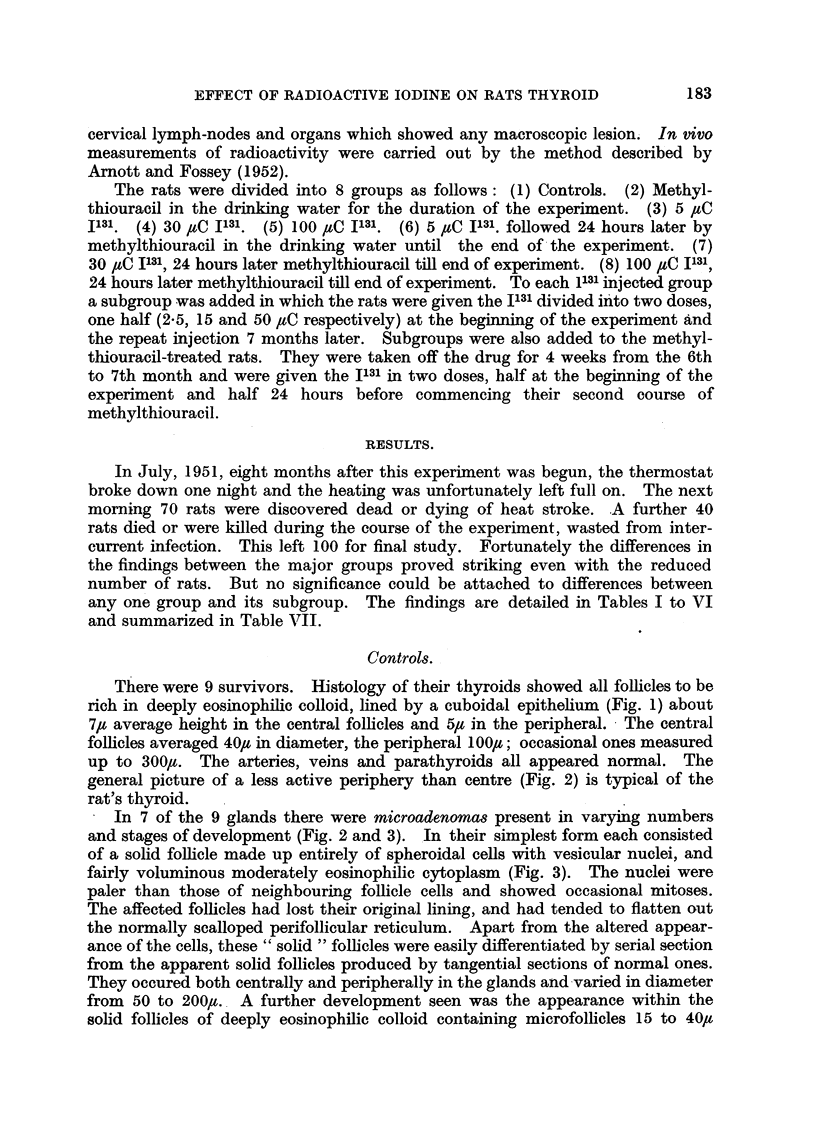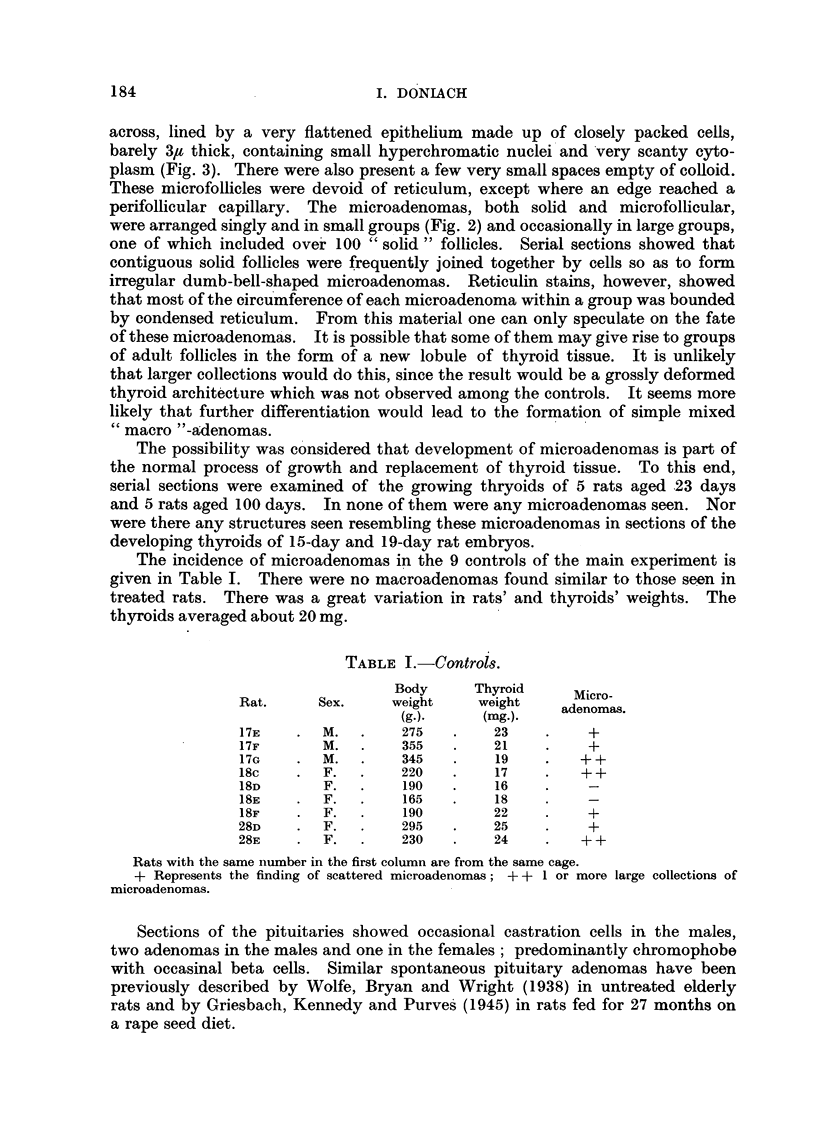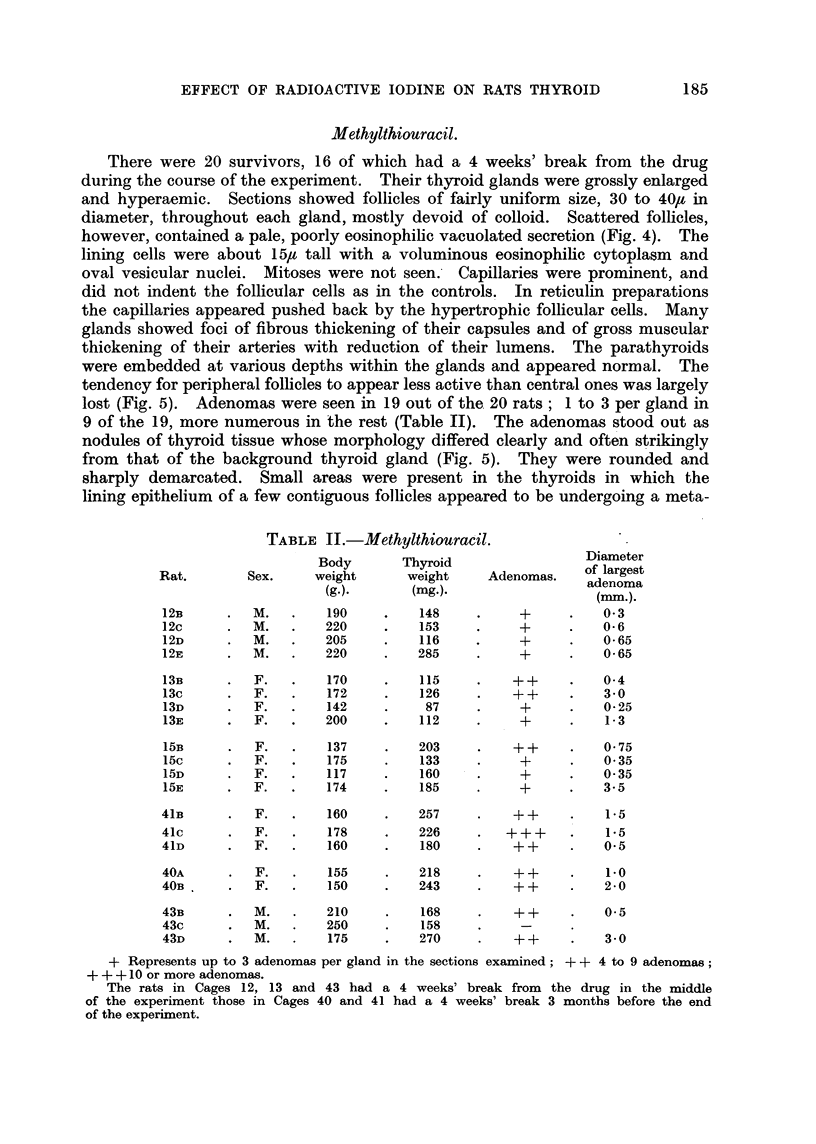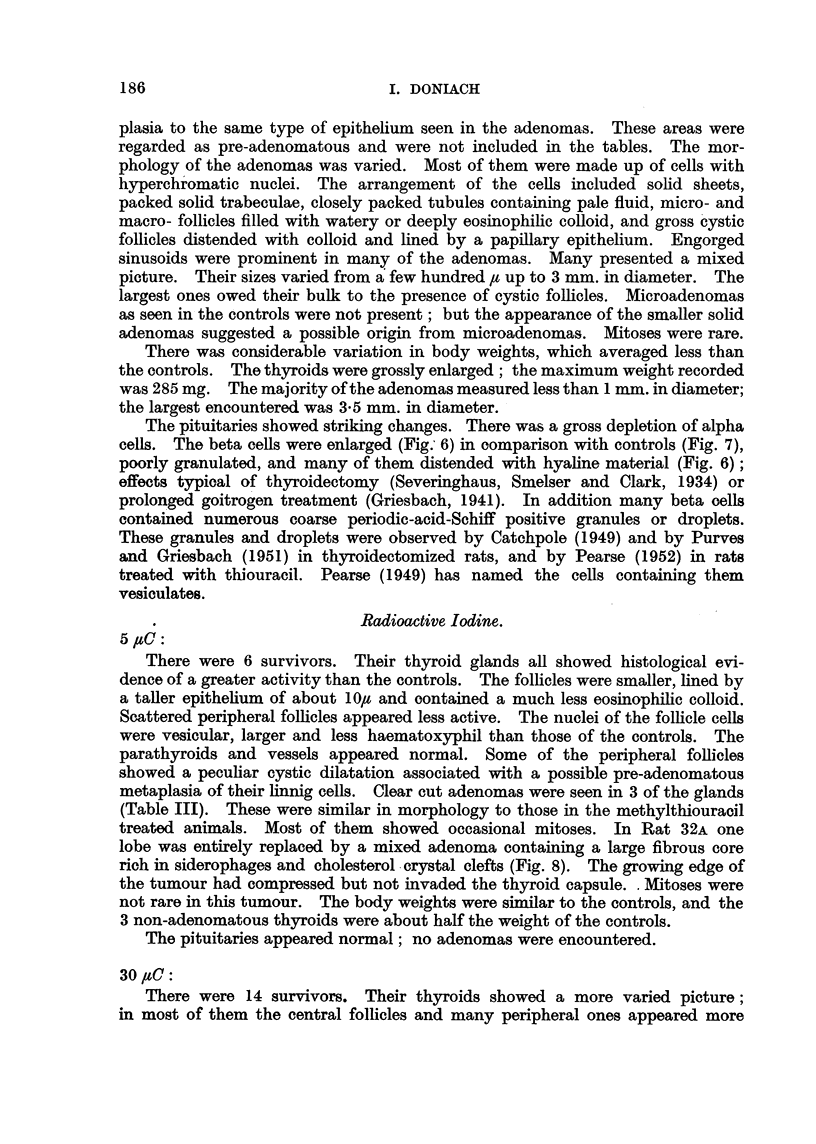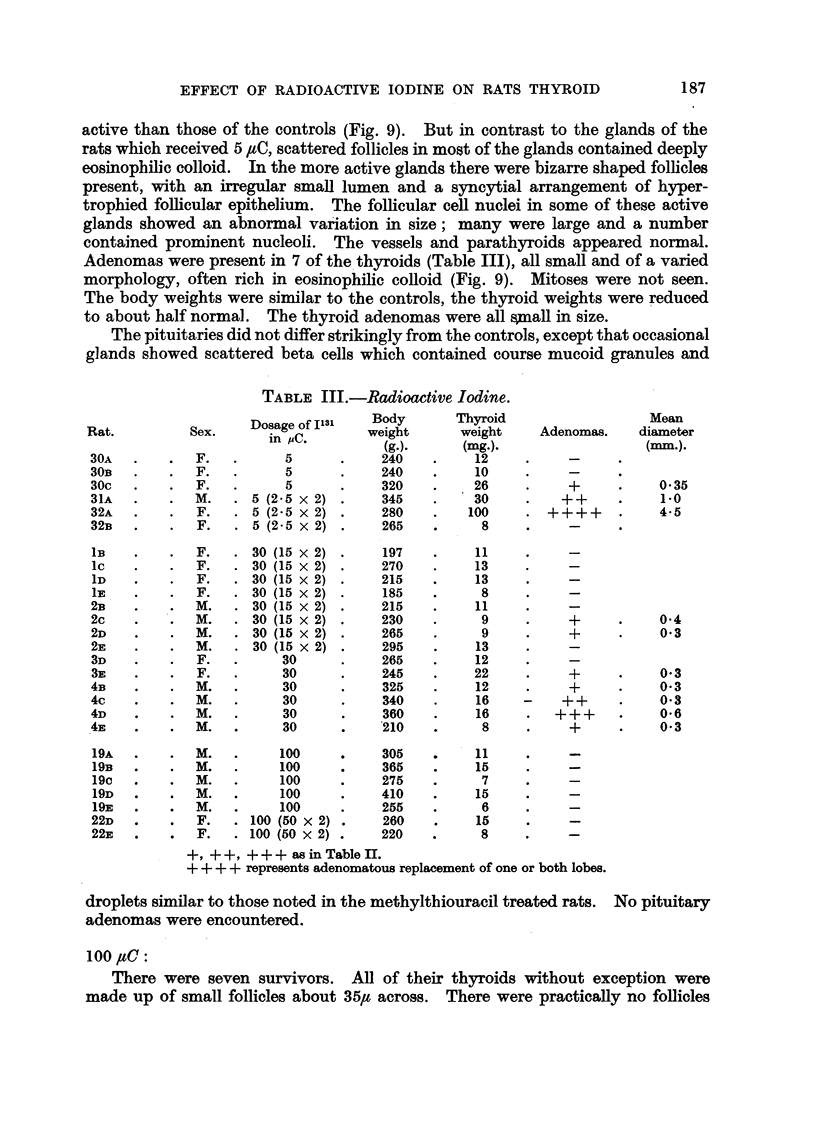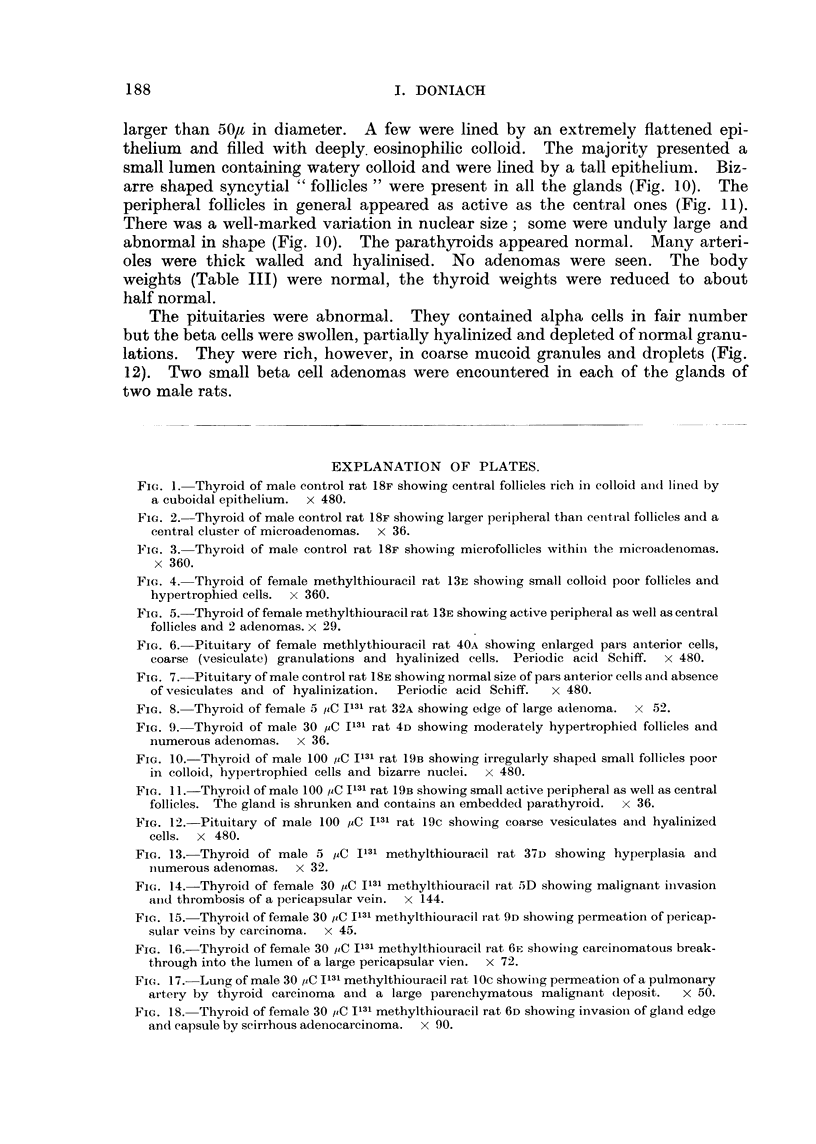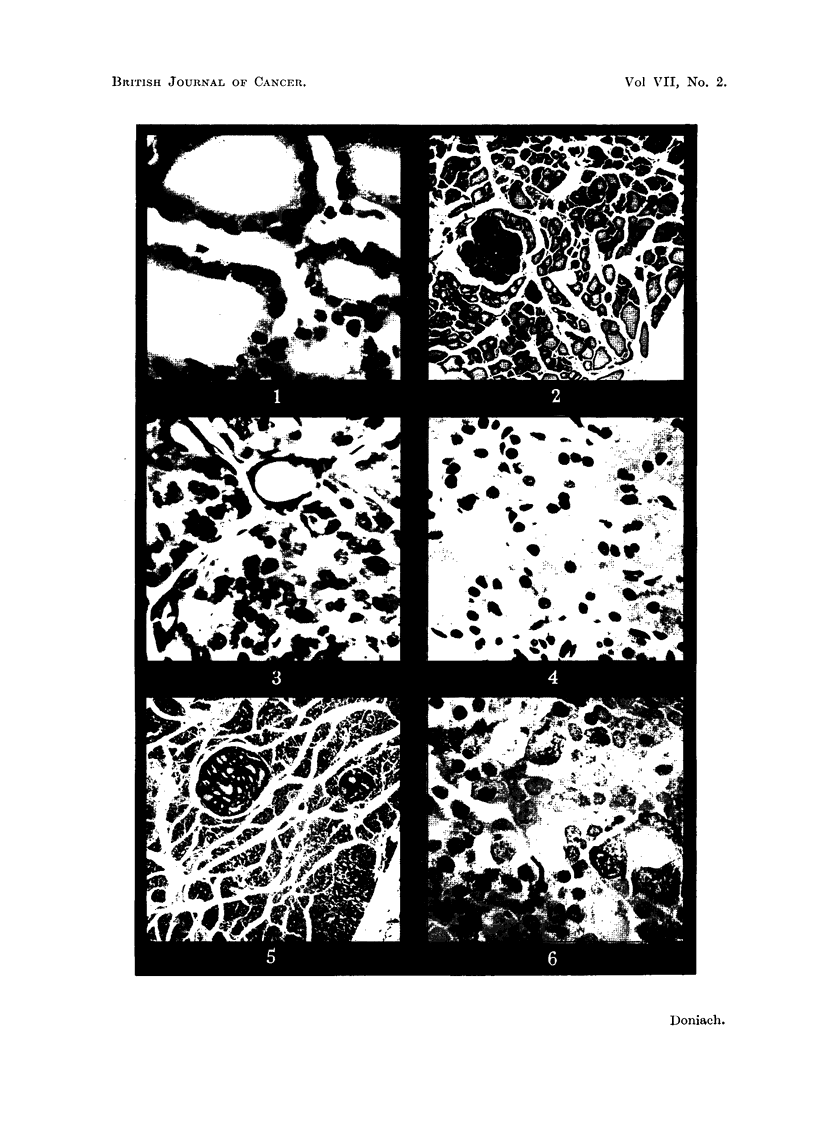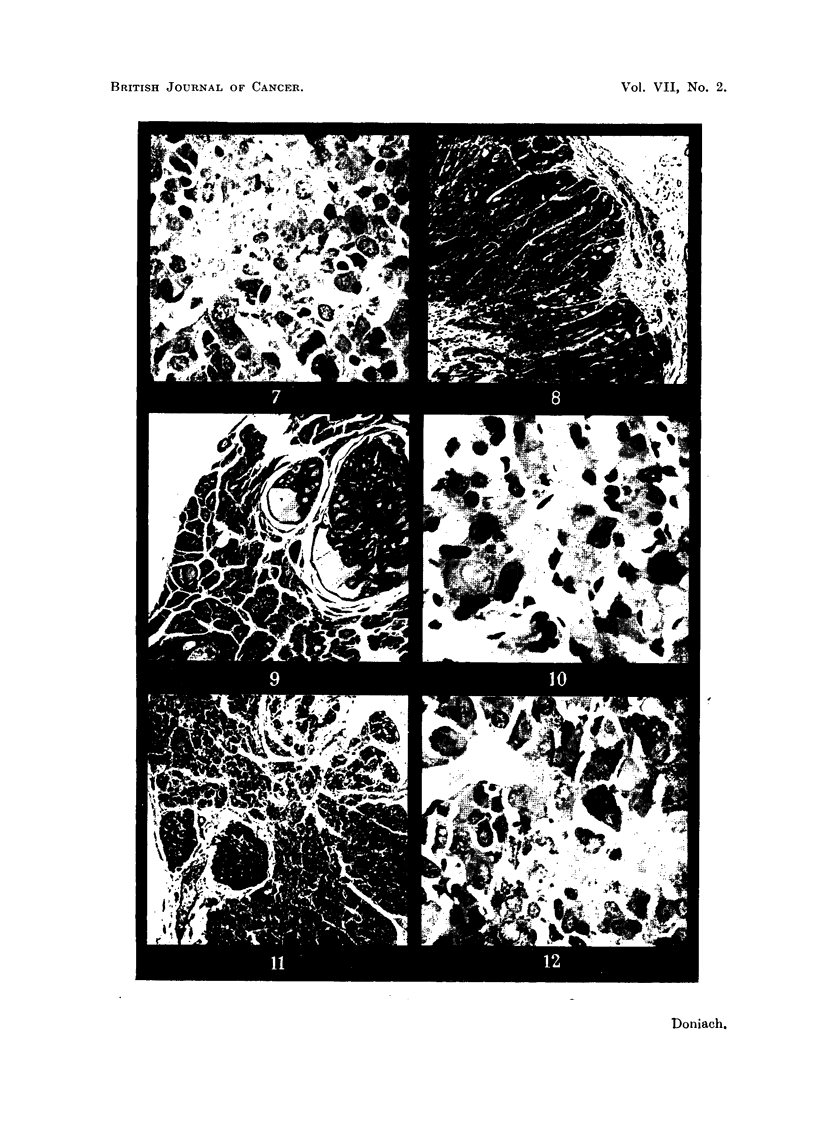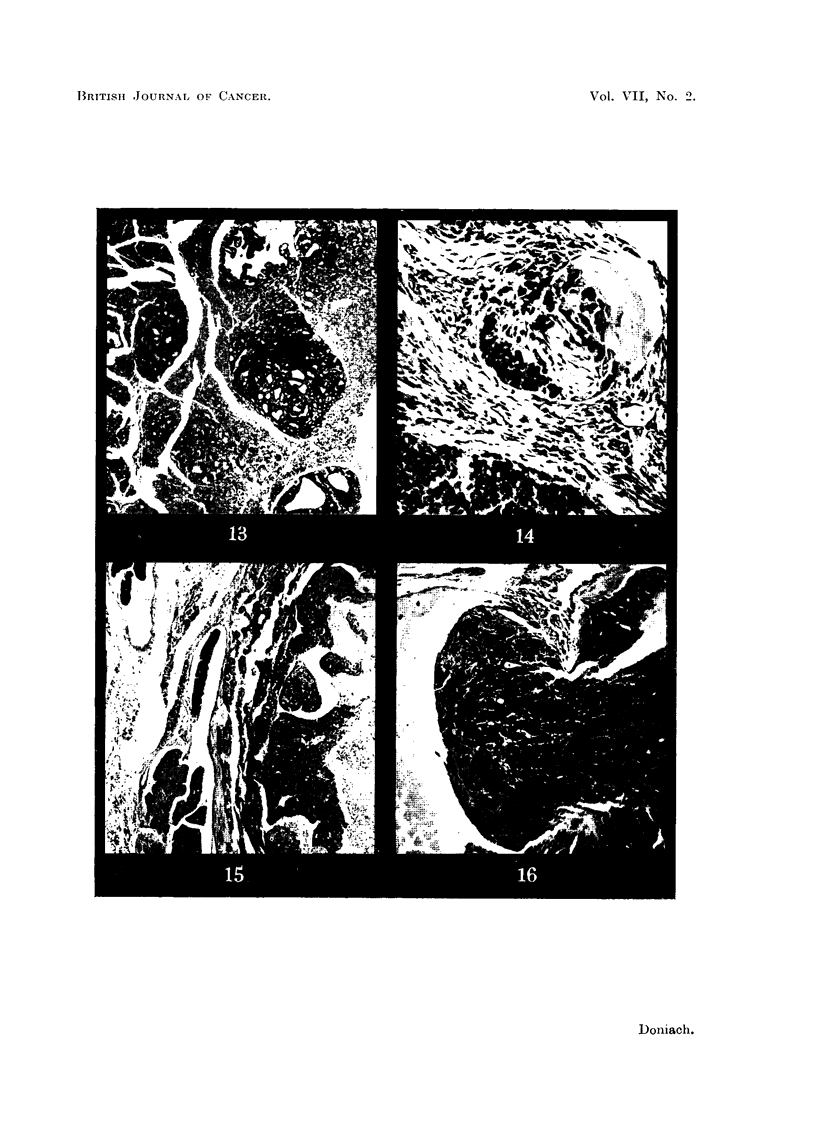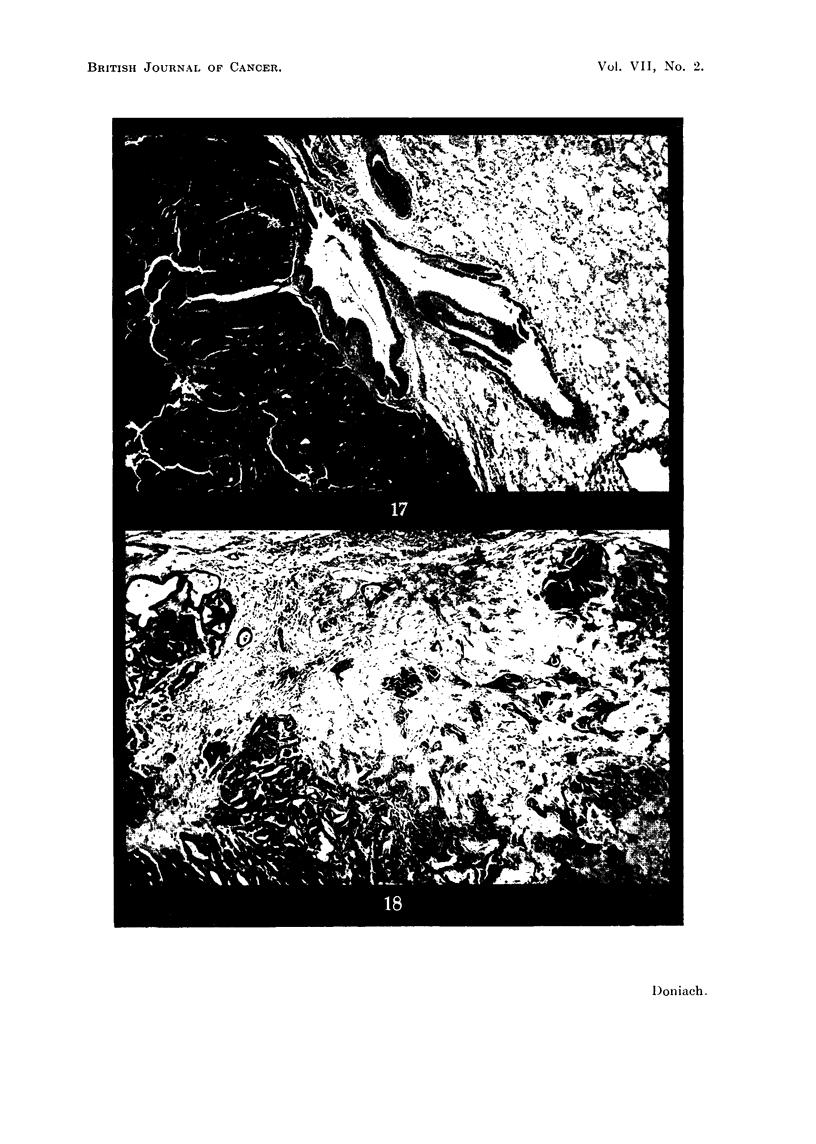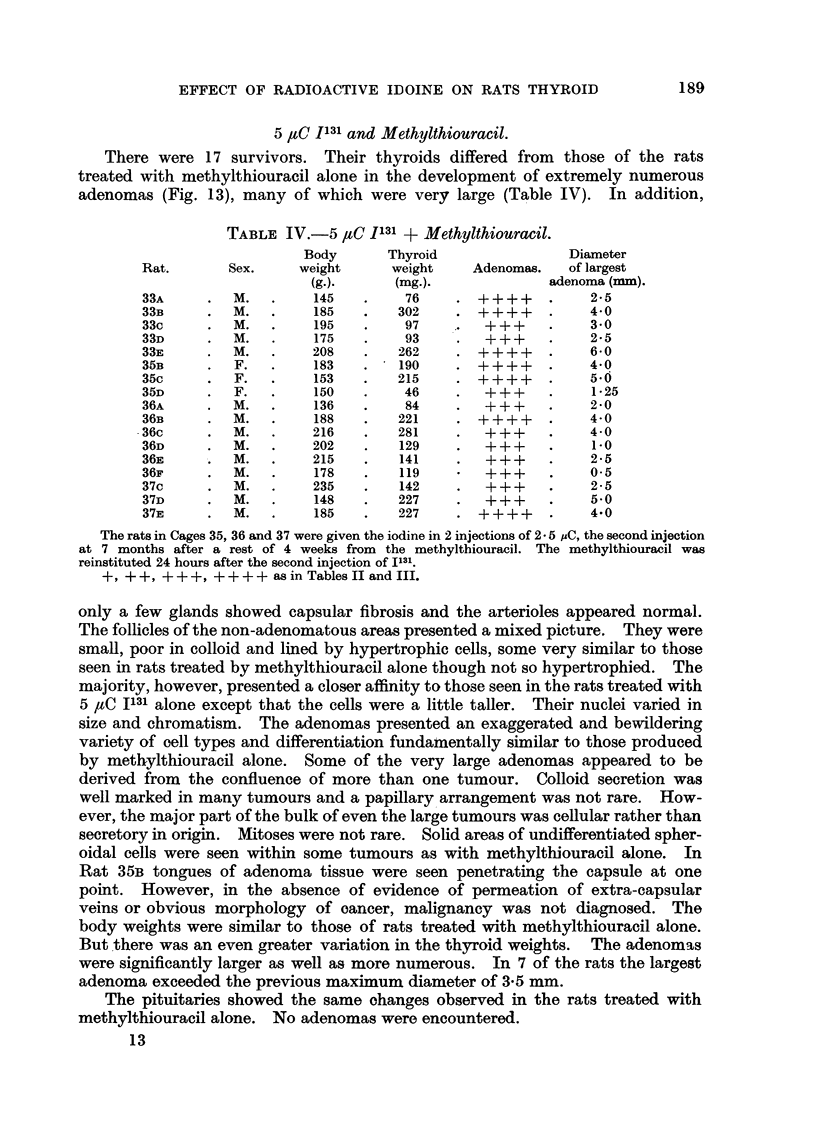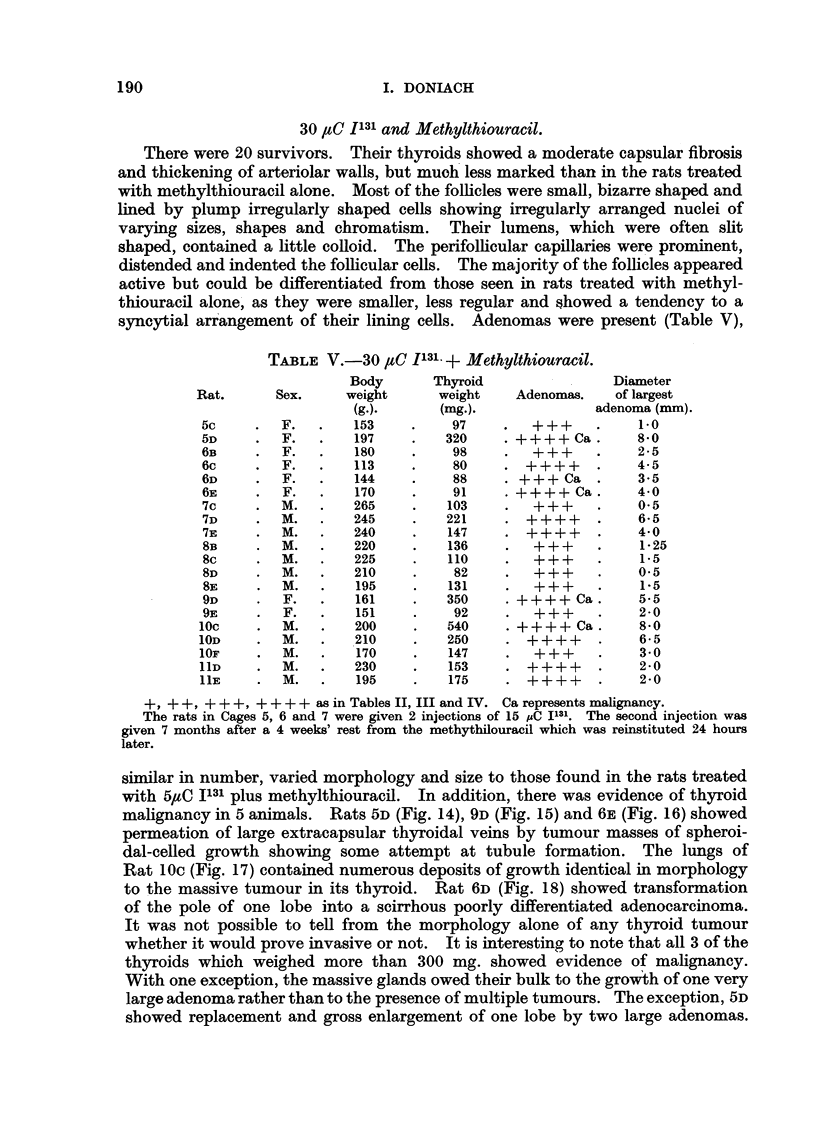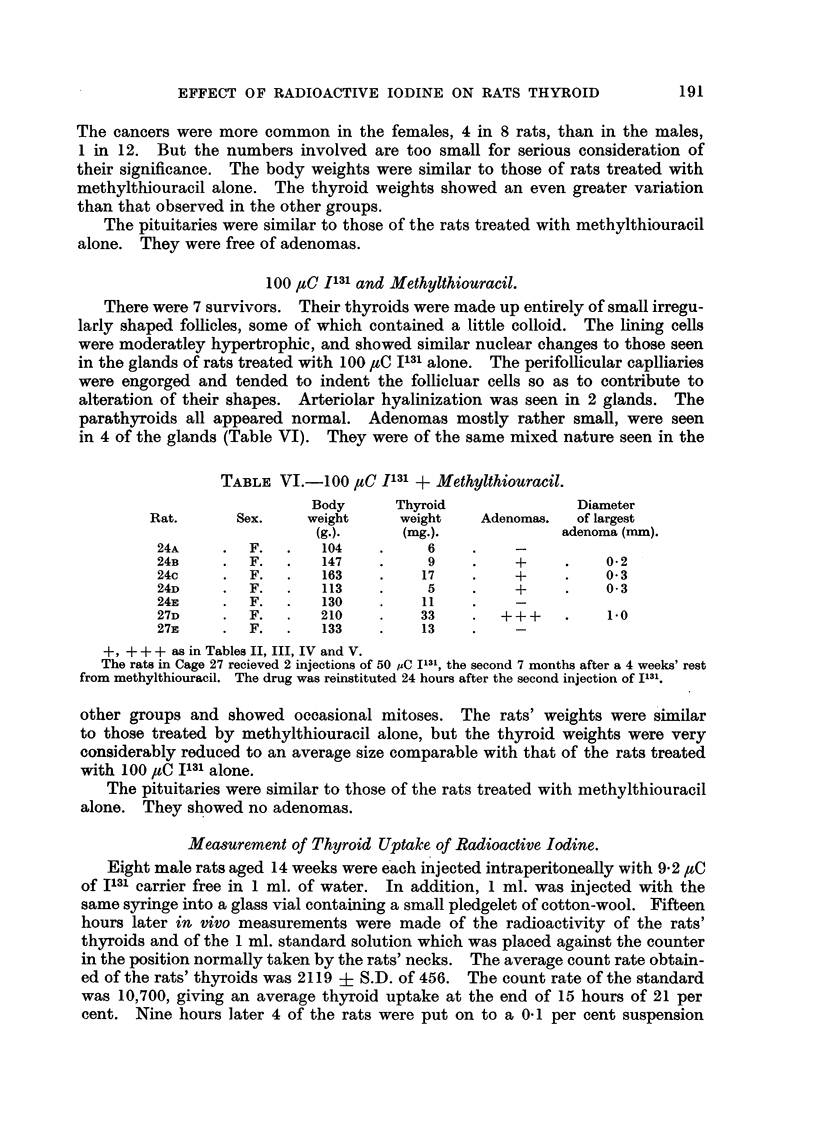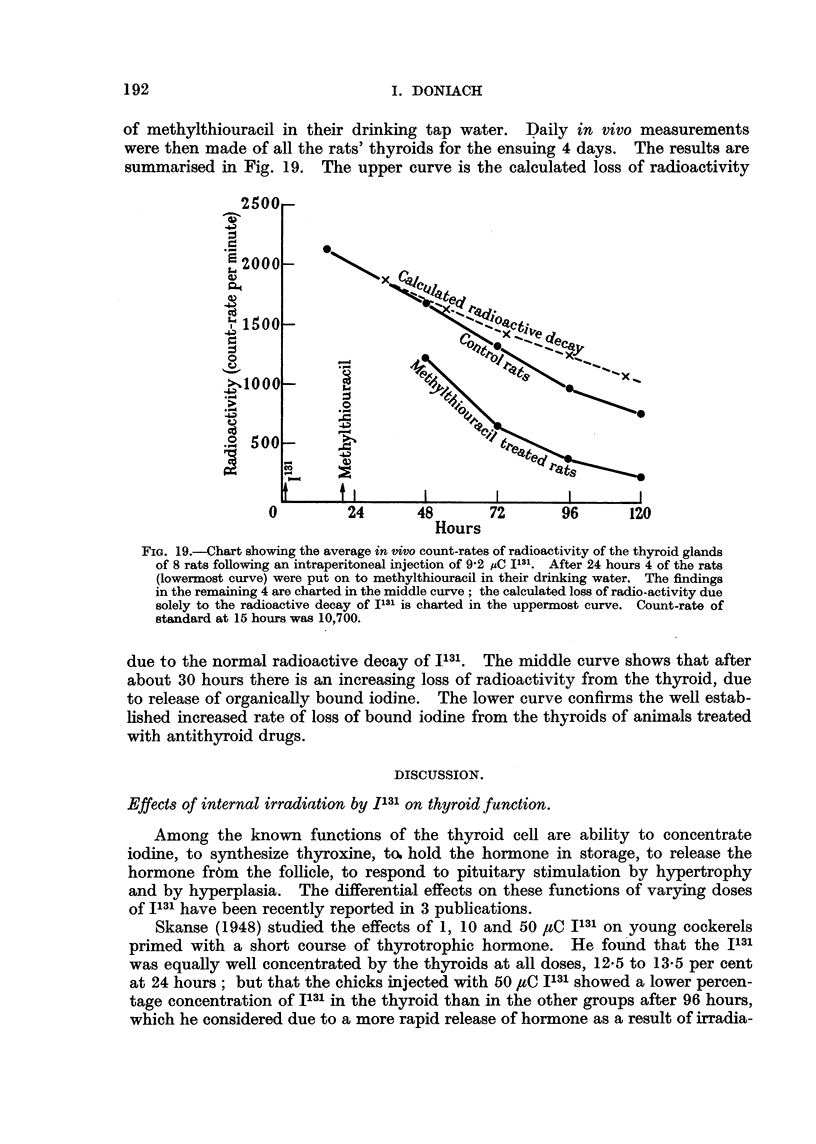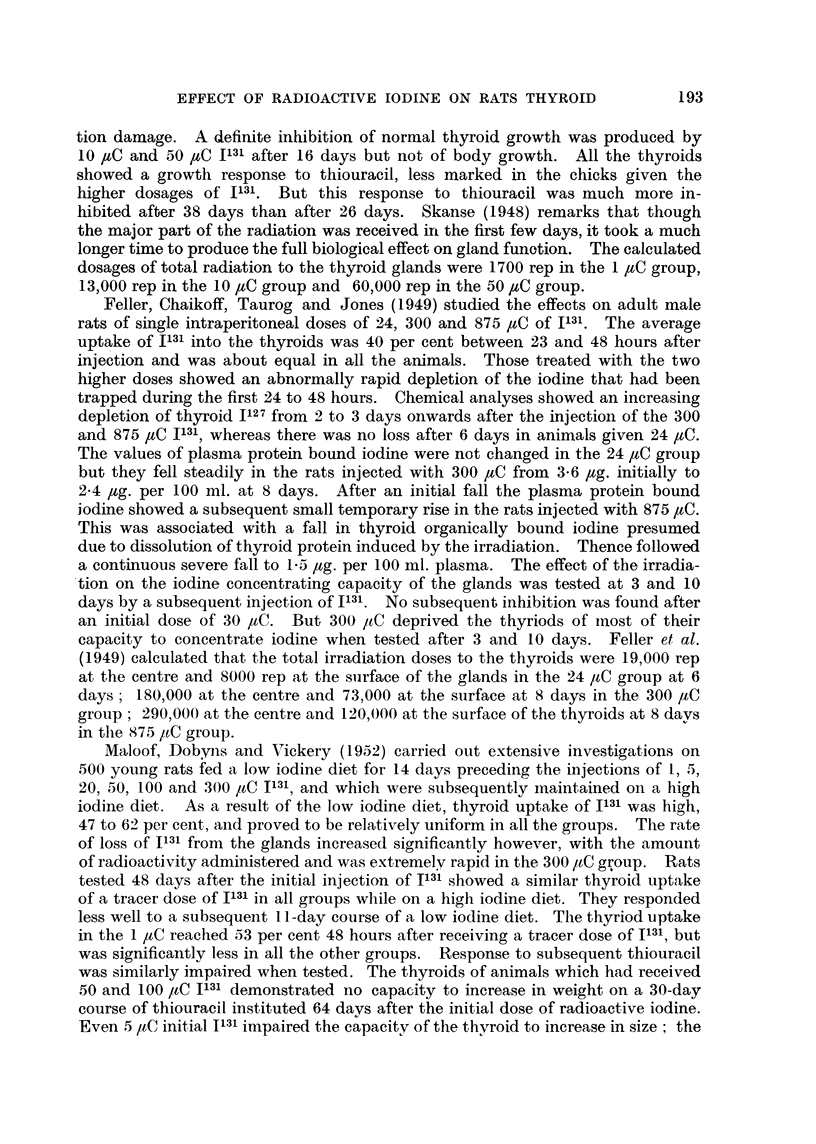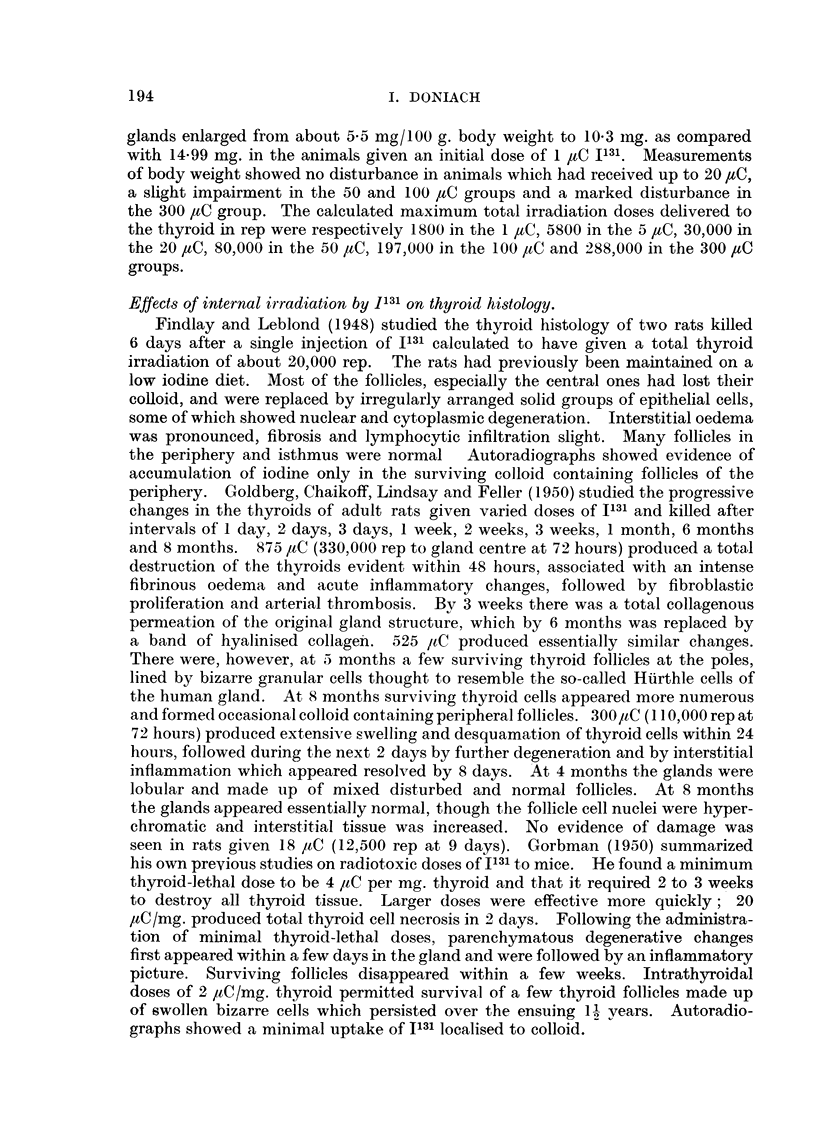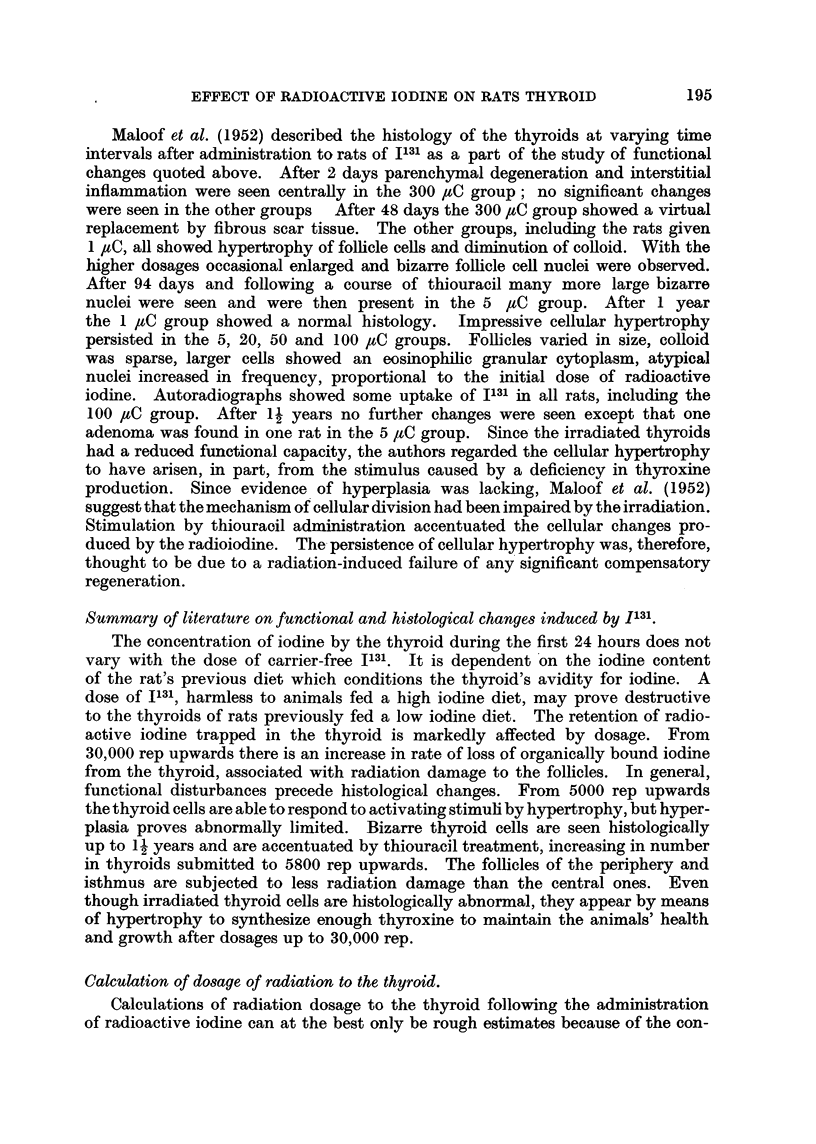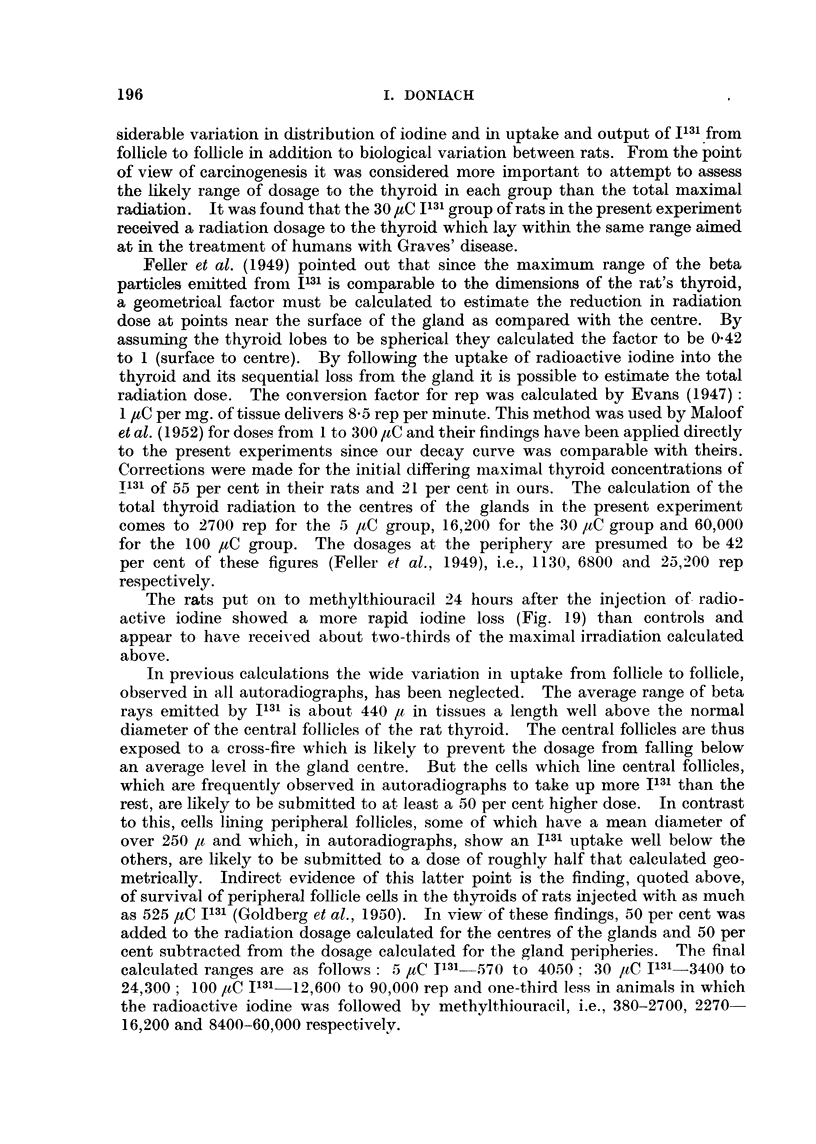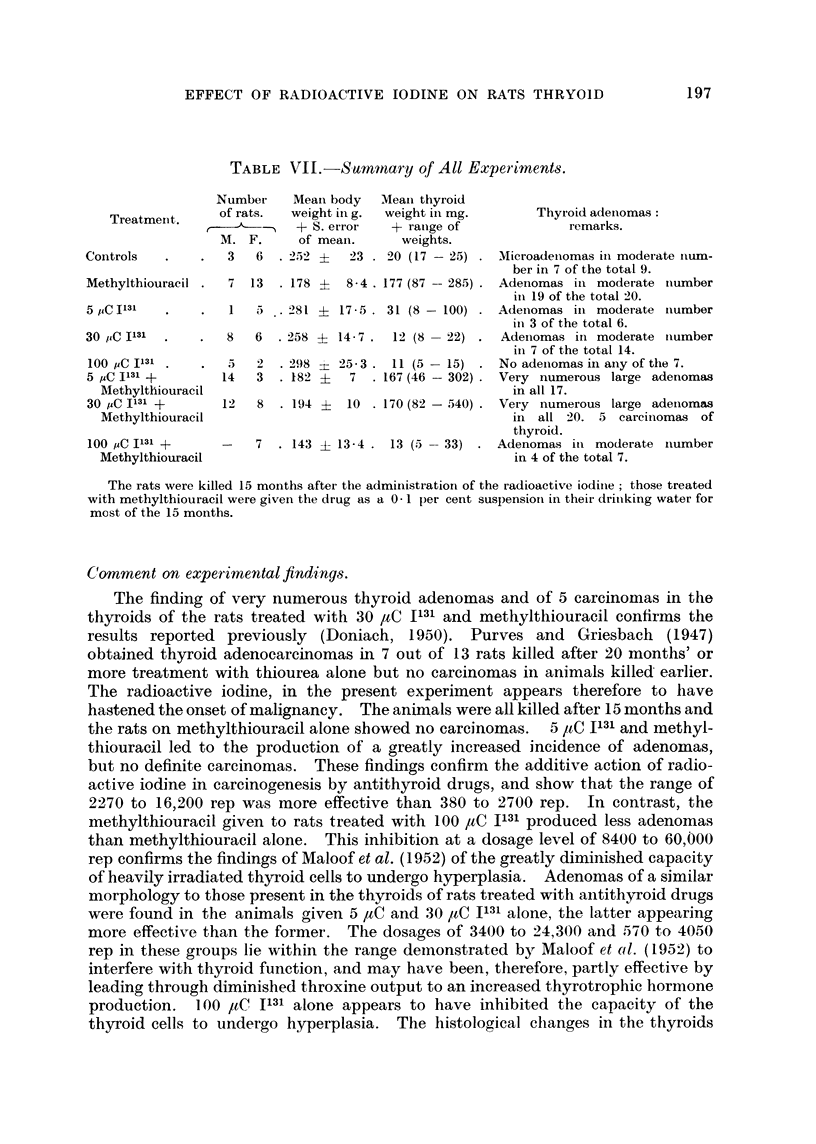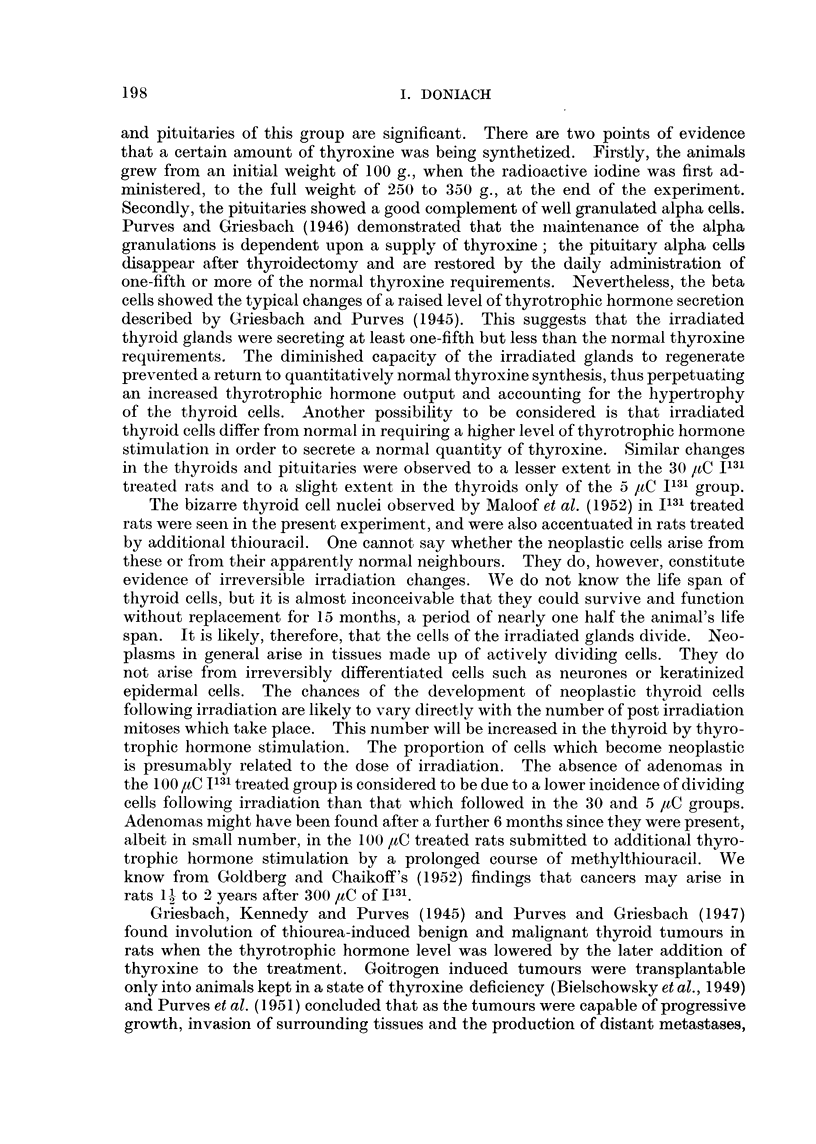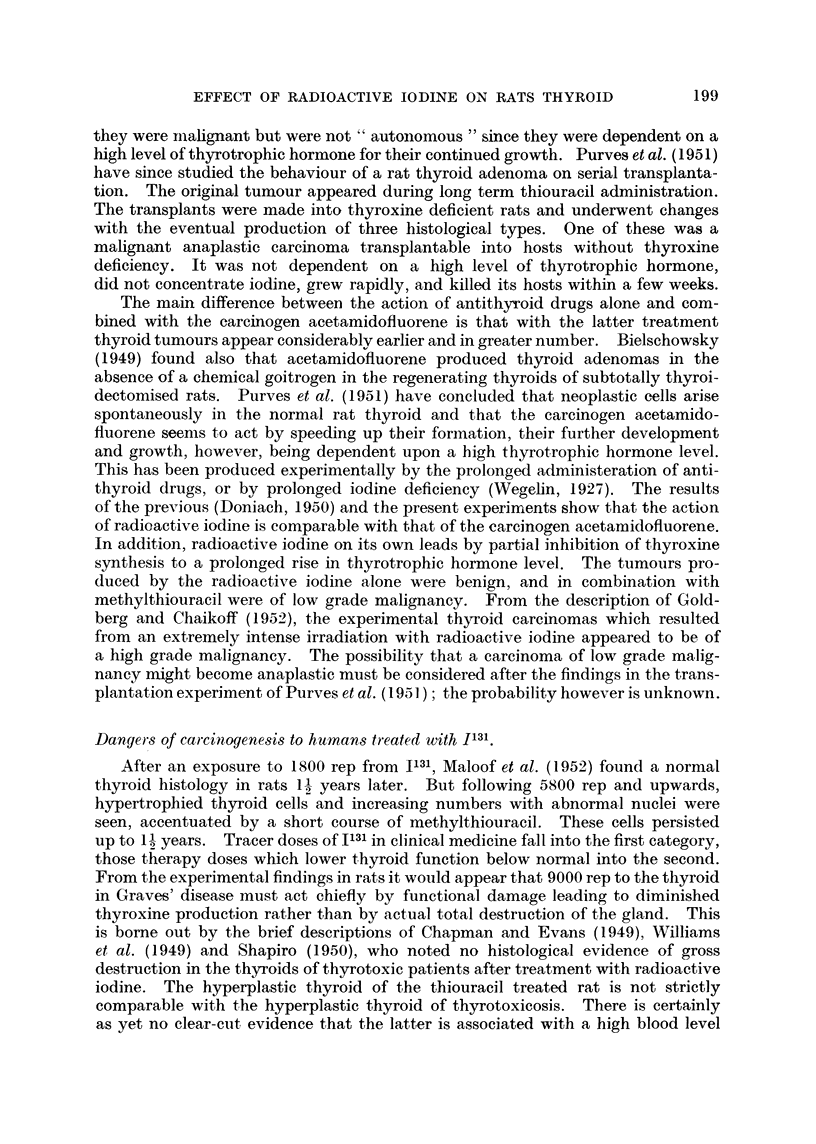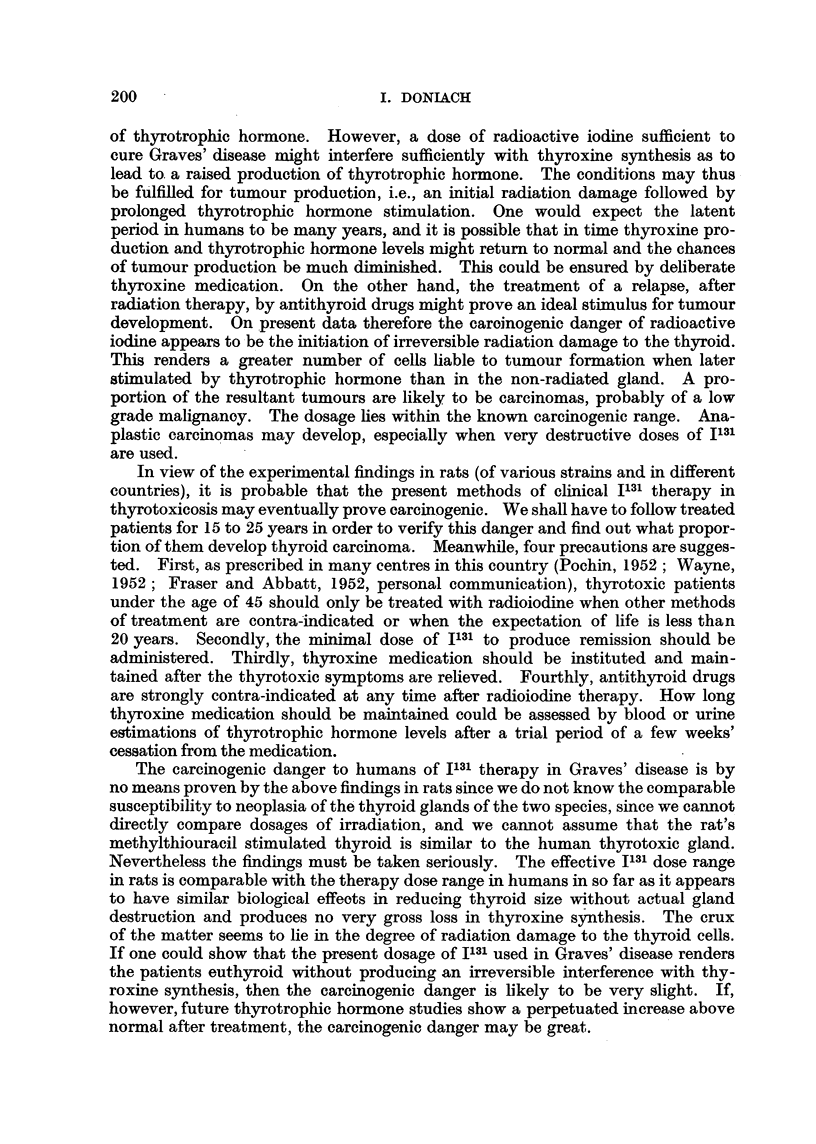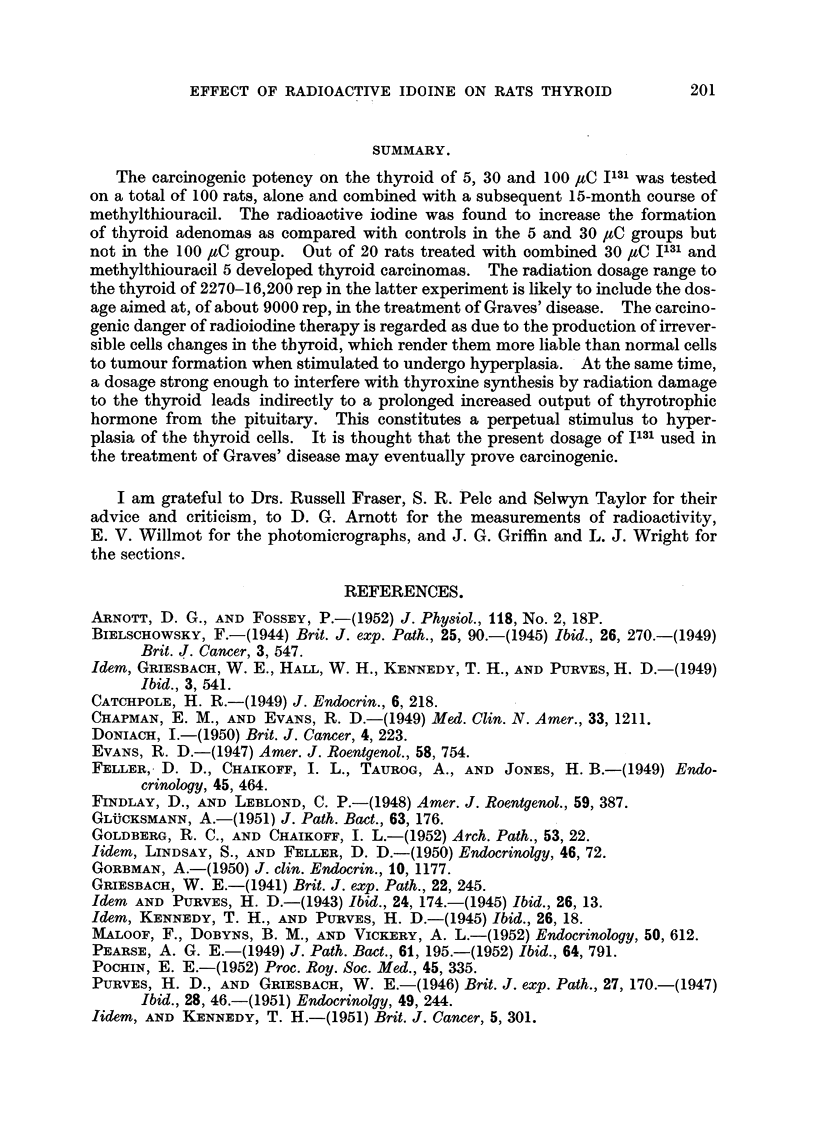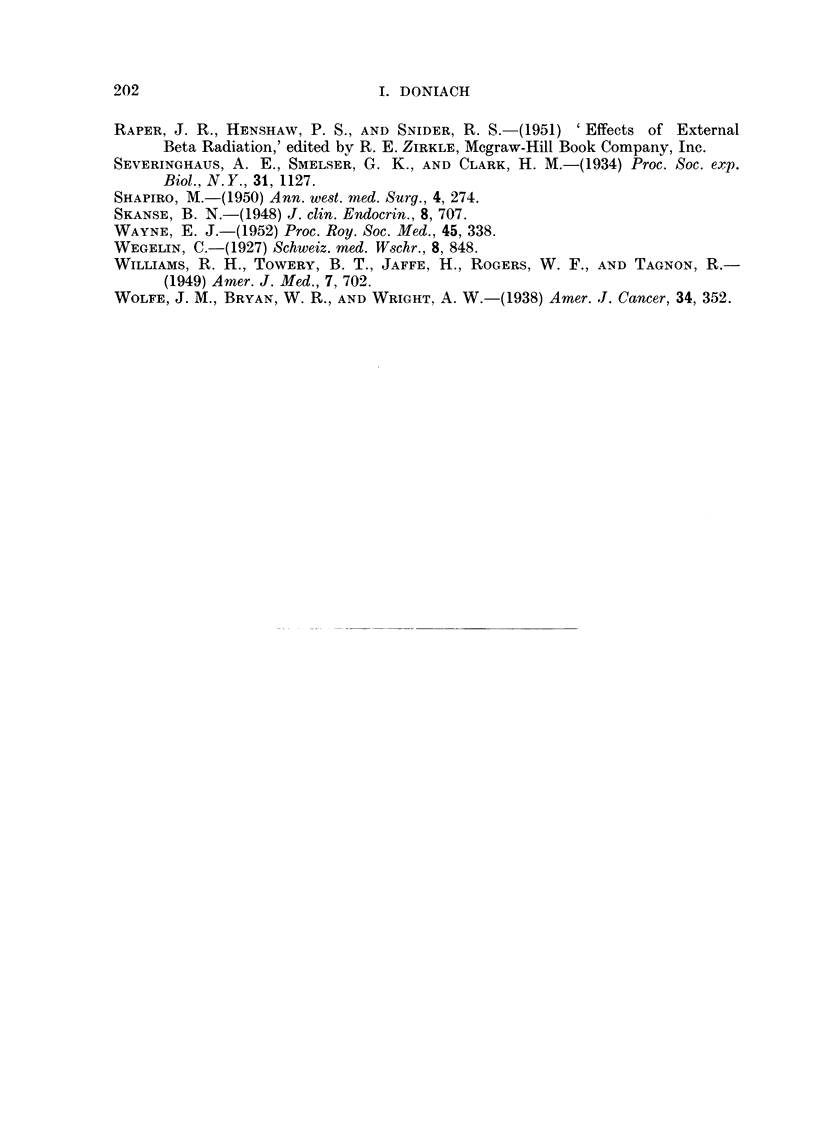# The Effect of Radioactive Iodine alone and in Combination with Methylthiouracil upon Tumour Production in the Rat's Thyroid Gland

**DOI:** 10.1038/bjc.1953.17

**Published:** 1953-06

**Authors:** I. Doniach

## Abstract

**Images:**


### THE EFFECT OF RADIOACTIVE IODINE ALONE AND IN COMBINATION WITH METHYLTHIOURACIL UPON TUMOUR PRODUCTION IN THE RAT'S THYROID GLAND.

I. DONIACH.

*From the Pathology Department, Postgraduate Medical School of London, W.12.*

Received for publication February 20, 1953.

THE increasing use of radioactive iodine in clinical medicine has made imperitive the experimental study of its possible carcinogenic action on the thyroid gland. Radioactive iodine, like stable iodine, is rapidly concentrated by the thyroid, bound to protein in its colloid in the synthesis of thyroxine, and then gradually released from the gland as part of the normally secreted hormone. During these processes the follicular cells of the thyroid are submitted to a course of ionizing irradiation from the $I^{131}$, which gives out $\beta$ and $\gamma$ rays. The energy absorbed is almost entirely derived from the $\beta$ rays. The duration of this irradiation is of the order of days, since the half-life of $I^{131}$ is 8 days and the effective half-life of intrafollicular hormone is also of the order of days in the normal human gland. The carcinogenic activity of ionizing radiations in general has been repeatedly demonstrated during the past 50 years. Recently, the effects of $\beta$ irradiation from newly developed sources have been studied. Raper, Henshaw and Snider (1951) reported that single doses of 4000 to 5000 rep (roentgens equivalent physical) of $\beta$ rays from a $P^{32}$ source induced skin tumours in rats. They also found that 13,400 rep given in daily exposures of 50 rep over a period of 348 days proved carcinogenic. Glücksmann (1951) found epitheliomas in 4 out of 24 mice in an area of skin exposed 14 months previously to 7900 rep given in 30 seconds by an electron beam.

It has proved necessary in the treatment of Graves' disease with radioactive iodine to administer an amount of $I^{131}$ calculated to give an overall irradiation to the thyroid gland of 8000 to 10,000 rep (Wayne, 1952) in order to produce a complete remission of the disease. This dosage lies within the range found to be carcinogenic to other animal tissues. But owing to the uneven distribution of radioactive iodine in the thyroid one cannot make a confident direct comparison, from the dosage point of view, between the animal experiments mentioned above and $I^{131}$ therapy of humans.

The mechanism of the experimental production of tumours of the thyroid by goitrogens has been greatly clarified by the New Zealand team of workers (Purves, Griesbach and Kennedy, 1951). They have shown by a series of elegant experiments that, in the rat, a long continued stimulation of the gland by prolonged excessive secretion of thyrotrophic hormone by the anterior hypophysis plays an essential role in the induction and progressive growth of these tumours. This was accomplished by the production of a chronic thyroxine deficiency with antithyroid drugs, since thyroxine deficiency however produced, leads to increased thyrotrophic hormone production (Griesbach and Purves, 1943).

In evaluating the possible danger of carcinogenesis by $I^{131}$ one must consider not only the direct action of $\beta$ rays on the cells, but also the indirect action of radiation damage, which leads to diminished thyroxine synthesis and a resultant increased thyrotrophic hormone production. A suitable dose of $I^{131}$ might therefore lead to a summation of two carcinogenic stimuli; a short exposure to $\beta$ irradiation and a long exposure to stimulation by the pituitary. The latter could be ensured experimentally by giving a prolonged course of antithyroid drug subsequent to a dose of $I^{131}$. Doniach (1950) found that 10 out of 16 rats, given 32 microcuries of $I^{131}$ (15,000 rep to the thyroid), developed adenomas. In a series of 5 rats given additional methylthiouracil for 14 months, the thyroids showed a striking increase in adenomas and in one instance a metastasizing carcinoma, in contrast to 16 rats tested for the same period with methylthiouracil alone, whose thyroids showed only occasional adenomas. This summating effect of a carcinogen with an antithyroid drug was first demonstrated by Bielschowsky (1944, 1945), who used the carcinogen acetamidofluorene combined with allylthiourea. Goldberg and Chaikoff (1952) have recently reported the development of poorly differentiated non-colloid-forming thyroid cancers in 7 out of 25 rats injected $1\frac{1}{2}$ to 2 years previously with 400 microcuries of $I^{131}$. The thyroids must have been exposed to the very destructive dosage of at least 150,000 rep. The authors attributed the cancer development entirely to beta irradiation, and not to thyrotrophic hormone stimulation.

The object of the experiments described below was to repeat previous investigations of the action of 30 microcuries of $I^{131}$ alone and combined with methylthiouracil in a larger series of rats, and to compare these results with the effects of 5 and of 100 microcuries. The findings are discussed in relation to various points enumerated in the introduction above, and an attempt is made to assess the dangers of carcinogenesis following the clinical usage of radioactive iodine.

### MATERIAL AND METHODS.

For the main experiment 210 rats of both sexes were used and a further 20 for subsidiary studies of dosage and thyroid histology. They were hooded rats of the Lister strain, fed on " Research (Rat) Cubes " with additional bread and greens. The 4-methyl-2-thiouracil was given as a saturated solution in the drinking tap water, made up once weekly by suspending 1 g. of compound in each litre of water. The radioactive iodine $I^{131}$ was injected intraperitoneally, carrier-free as iodide in 1 ml. of water. The animals averaged 10 weeks in age at the beginning of the experiment and were killed by coal gas 15 *months* later. The trachea and thyroid attached were fixed in Helly's fluid; the thyroid was then dissected off and weighed to the nearest milligram. The thyroids were embedded in wax, all glands which weighed 30 mg. or less were serially sectioned at $8\mu$, mounted in ribbons of about 12 sections per slide, and every alternate slide was stained by haemalum and eosin. Glands weighing more than 30 mg. were sectioned at 6 levels, so arranged as to traverse the whole thickness of the thyroid at regular intervals. Three sections cut at each level at $5\mu$ were mounted and one of them stained by haemalum and eosin. The spare sections in general were used for extra stains. The pituitaries were fixed in Helly and stained by haemalum and eosin and by the trichrome periodic-acid-Schiff method of Pearse (1949). Random sections were also taken in a number of rats of the lungs, adrenals, cervical lymph-nodes and organs which showed any macroscopic lesion. *In vivo* measurements of radioactivity were carried out by the method described by Arnott and Fossey (1952).

The rats were divided into 8 groups as follows: (1) Controls. (2) Methylthiouracil in the drinking water for the duration of the experiment. (3) 5 $\mu$C $I^{131}$. (4) 30 $\mu$C $I^{131}$. (5) 100 $\mu$C $I^{131}$. (6) 5 $\mu$C $I^{131}$. followed 24 hours later by methylthiouracil in the drinking water until the end of the experiment. (7) 30 $\mu$C $I^{131}$, 24 hours later methylthiouracil till end of experiment. (8) 100 $\mu$C $I^{131}$, 24 hours later methylthiouracil till end of experiment. To each $I^{131}$ injected group a subgroup was added in which the rats were given the $I^{131}$ divided into two doses, one half (2·5, 15 and 50 $\mu$C respectively) at the beginning of the experiment and the repeat injection 7 months later. Subgroups were also added to the methylthiouracil-treated rats. They were taken off the drug for 4 weeks from the 6th to 7th month and were given the $I^{131}$ in two doses, half at the beginning of the experiment and half 24 hours before commencing their second course of methylthiouracil.

### RESULTS.

In July, 1951, eight months after this experiment was begun, the thermostat broke down one night and the heating was unfortunately left full on. The next morning 70 rats were discovered dead or dying of heat stroke. A further 40 rats died or were killed during the course of the experiment, wasted from intercurrent infection. This left 100 for final study. Fortunately the differences in the findings between the major groups proved striking even with the reduced number of rats. But no significance could be attached to differences between any one group and its subgroup. The findings are detailed in Tables I to VI and summarized in Table VII.

### *Controls.*

There were 9 survivors. Histology of their thyroids showed all follicles to be rich in deeply eosinophilic colloid, lined by a cuboidal epithelium (Fig. 1) about 7$\mu$ average height in the central follicles and 5$\mu$ in the peripheral. The central follicles averaged 40$\mu$ in diameter, the peripheral 100$\mu$; occasional ones measured up to 300$\mu$. The arteries, veins and parathyroids all appeared normal. The general picture of a less active periphery than centre (Fig. 2) is typical of the rat's thyroid.

In 7 of the 9 glands there were *microadenomas* present in varying numbers and stages of development (Fig. 2 and 3). In their simplest form each consisted of a solid follicle made up entirely of spheroidal cells with vesicular nuclei, and fairly voluminous moderately eosinophilic cytoplasm (Fig. 3). The nuclei were paler than those of neighbouring follicle cells and showed occasional mitoses. The affected follicles had lost their original lining, and had tended to flatten out the normally scalloped perifollicular reticulum. Apart from the altered appearance of the cells, these "solid" follicles were easily differentiated by serial section from the apparent solid follicles produced by tangential sections of normal ones. They occured both centrally and peripherally in the glands and varied in diameter from 50 to 200$\mu$. A further development seen was the appearance within the solid follicles of deeply eosinophilic colloid containing microfollicles 15 to 40$\mu$

across, lined by a very flattened epithelium made up of closely packed cells, barely 3$\mu$ thick, containing small hyperchromatic nuclei and very scanty cytoplasm (Fig. 3). There were also present a few very small spaces empty of colloid. These microfollicles were devoid of reticulum, except where an edge reached a perifollicular capillary. The microadenomas, both solid and microfollicular, were arranged singly and in small groups (Fig. 2) and occasionally in large groups, one of which included over 100 " solid " follicles. Serial sections showed that contiguous solid follicles were frequently joined together by cells so as to form irregular dumb-bell-shaped microadenomas. Reticulin stains, however, showed that most of the circumference of each microadenoma within a group was bounded by condensed reticulum. From this material one can only speculate on the fate of these microadenomas. It is possible that some of them may give rise to groups of adult follicles in the form of a new lobule of thyroid tissue. It is unlikely that larger collections would do this, since the result would be a grossly deformed thyroid architecture which was not observed among the controls. It seems more likely that further differentiation would lead to the formation of simple mixed " macro "-adenomas.

The possibility was considered that development of microadenomas is part of the normal process of growth and replacement of thyroid tissue. To this end, serial sections were examined of the growing thryoids of 5 rats aged 23 days and 5 rats aged 100 days. In none of them were any microadenomas seen. Nor were there any structures seen resembling these microadenomas in sections of the developing thyroids of 15-day and 19-day rat embryos.

The incidence of microadenomas in the 9 controls of the main experiment is given in Table I. There were no macroadenomas found similar to those seen in treated rats. There was a great variation in rats' and thyroids' weights. The thyroids averaged about 20 mg.

TABLE I.—*Controls.*

| Rat. | Sex. | Body weight (g.). | Thyroid weight (mg.). | Micro-adenomas. |
|---|---|---|---|---|
| 17E | M. | 275 | 23 | + |
| 17F | M. | 355 | 21 | + |
| 17G | M. | 345 | 19 | ++ |
| 18C | F. | 220 | 17 | ++ |
| 18D | F. | 190 | 16 | − |
| 18E | F. | 165 | 18 | − |
| 18F | F. | 190 | 22 | + |
| 28D | F. | 295 | 25 | + |
| 28E | F. | 230 | 24 | ++ |

Rats with the same number in the first column are from the same cage.

+ Represents the finding of scattered microadenomas; ++ 1 or more large collections of microadenomas.

Sections of the pituitaries showed occasional castration cells in the males, two adenomas in the males and one in the females; predominantly chromophobe with occasinal beta cells. Similar spontaneous pituitary adenomas have been previously described by Wolfe, Bryan and Wright (1938) in untreated elderly rats and by Griesbach, Kennedy and Purves (1945) in rats fed for 27 months on a rape seed diet.

*Methylthiouracil.*

There were 20 survivors, 16 of which had a 4 weeks' break from the drug during the course of the experiment. Their thyroid glands were grossly enlarged and hyperaemic. Sections showed follicles of fairly uniform size, 30 to 40$\mu$ in diameter, throughout each gland, mostly devoid of colloid. Scattered follicles, however, contained a pale, poorly eosinophilic vacuolated secretion (Fig. 4). The lining cells were about 15$\mu$ tall with a voluminous eosinophilic cytoplasm and oval vesicular nuclei. Mitoses were not seen. Capillaries were prominent, and did not indent the follicular cells as in the controls. In reticulin preparations the capillaries appeared pushed back by the hypertrophic follicular cells. Many glands showed foci of fibrous thickening of their capsules and of gross muscular thickening of their arteries with reduction of their lumens. The parathyroids were embedded at various depths within the glands and appeared normal. The tendency for peripheral follicles to appear less active than central ones was largely lost (Fig. 5). Adenomas were seen in 19 out of the 20 rats ; 1 to 3 per gland in 9 of the 19, more numerous in the rest (Table II). The adenomas stood out as nodules of thyroid tissue whose morphology differed clearly and often strikingly from that of the background thyroid gland (Fig. 5). They were rounded and sharply demarcated. Small areas were present in the thyroids in which the lining epithelium of a few contiguous follicles appeared to be undergoing a meta-

TABLE II.—*Methylthiouracil.*

| Rat. | Sex. | Body weight (g.). | Thyroid weight (mg.). | Adenomas. | Diameter of largest adenoma (mm.). |
|---|---|---|---|---|---|
| 12B | M. | 190 | 148 | + | 0·3 |
| 12C | M. | 220 | 153 | + | 0·6 |
| 12D | M. | 205 | 116 | + | 0·65 |
| 12E | M. | 220 | 285 | + | 0·65 |
| 13B | F. | 170 | 115 | ++ | 0·4 |
| 13C | F. | 172 | 126 | ++ | 3·0 |
| 13D | F. | 142 | 87 | + | 0·25 |
| 13E | F. | 200 | 112 | + | 1·3 |
| 15B | F. | 137 | 203 | ++ | 0·75 |
| 15C | F. | 175 | 133 | + | 0·35 |
| 15D | F. | 117 | 160 | + | 0·35 |
| 15E | F. | 174 | 185 | + | 3·5 |
| 41B | F. | 160 | 257 | ++ | 1·5 |
| 41C | F. | 178 | 226 | +++ | 1·5 |
| 41D | F. | 160 | 180 | ++ | 0·5 |
| 40A | F. | 155 | 218 | ++ | 1·0 |
| 40B | F. | 150 | 243 | ++ | 2·0 |
| 43B | M. | 210 | 168 | ++ | 0·5 |
| 43C | M. | 250 | 158 | — | |
| 43D | M. | 175 | 270 | ++ | 3·0 |

+ Represents up to 3 adenomas per gland in the sections examined ; ++ 4 to 9 adenomas ; +++10 or more adenomas.

The rats in Cages 12, 13 and 43 had a 4 weeks' break from the drug in the middle of the experiment those in Cages 40 and 41 had a 4 weeks' break 3 months before the end of the experiment.

plasia to the same type of epithelium seen in the adenomas. These areas were regarded as pre-adenomatous and were not included in the tables. The morphology of the adenomas was varied. Most of them were made up of cells with hyperchromatic nuclei. The arrangement of the cells included solid sheets, packed solid trabeculae, closely packed tubules containing pale fluid, micro- and macro- follicles filled with watery or deeply eosinophilic colloid, and gross cystic follicles distended with colloid and lined by a papillary epithelium. Engorged sinusoids were prominent in many of the adenomas. Many presented a mixed picture. Their sizes varied from a few hundred $\mu$ up to 3 mm. in diameter. The largest ones owed their bulk to the presence of cystic follicles. Microadenomas as seen in the controls were not present ; but the appearance of the smaller solid adenomas suggested a possible origin from microadenomas. Mitoses were rare.

There was considerable variation in body weights, which averaged less than the controls. The thyroids were grossly enlarged ; the maximum weight recorded was 285 mg. The majority of the adenomas measured less than 1 mm. in diameter; the largest encountered was 3·5 mm. in diameter.

The pituitaries showed striking changes. There was a gross depletion of alpha cells. The beta cells were enlarged (Fig. 6) in comparison with controls (Fig. 7), poorly granulated, and many of them distended with hyaline material (Fig. 6) ; effects typical of thyroidectomy (Severinghaus, Smelser and Clark, 1934) or prolonged goitrogen treatment (Griesbach, 1941). In addition many beta cells contained numerous coarse periodic-acid-Schiff positive granules or droplets. These granules and droplets were observed by Catchpole (1949) and by Purves and Griesbach (1951) in thyroidectomized rats, and by Pearse (1952) in rats treated with thiouracil. Pearse (1949) has named the cells containing them vesiculates.

*Radioactive Iodine.*

5 $\mu C$ :

There were 6 survivors. Their thyroid glands all showed histological evidence of a greater activity than the controls. The follicles were smaller, lined by a taller epithelium of about 10$\mu$ and contained a much less eosinophilic colloid. Scattered peripheral follicles appeared less active. The nuclei of the follicle cells were vesicular, larger and less haematoxyphil than those of the controls. The parathyroids and vessels appeared normal. Some of the peripheral follicles showed a peculiar cystic dilatation associated with a possible pre-adenomatous metaplasia of their linnig cells. Clear cut adenomas were seen in 3 of the glands (Table III). These were similar in morphology to those in the methylthiouracil treated animals. Most of them showed occasional mitoses. In Rat 32A one lobe was entirely replaced by a mixed adenoma containing a large fibrous core rich in siderophages and cholesterol crystal clefts (Fig. 8). The growing edge of the tumour had compressed but not invaded the thyroid capsule. Mitoses were not rare in this tumour. The body weights were similar to the controls, and the 3 non-adenomatous thyroids were about half the weight of the controls.

The pituitaries appeared normal ; no adenomas were encountered.

30 $\mu C$ :

There were 14 survivors. Their thyroids showed a more varied picture ; in most of them the central follicles and many peripheral ones appeared more active than those of the controls (Fig. 9). But in contrast to the glands of the rats which received 5 $\mu$C, scattered follicles in most of the glands contained deeply eosinophilic colloid. In the more active glands there were bizarre shaped follicles present, with an irregular small lumen and a syncytial arrangement of hypertrophied follicular epithelium. The follicular cell nuclei in some of these active glands showed an abnormal variation in size; many were large and a number contained prominent nucleoli. The vessels and parathyroids appeared normal. Adenomas were present in 7 of the thyroids (Table III), all small and of a varied morphology, often rich in eosinophilic colloid (Fig. 9). Mitoses were not seen. The body weights were similar to the controls, the thyroid weights were reduced to about half normal. The thyroid adenomas were all small in size.

The pituitaries did not differ strikingly from the controls, except that occasional glands showed scattered beta cells which contained course mucoid granules and

TABLE III.—*Radioactive Iodine.*

| Rat. | Sex. | Dosage of $I^{131}$ in $\mu$C. | Body weight (g.). | Thyroid weight (mg.). | Adenomas. | Mean diameter (mm.). |
|---|---|---|---|---|---|---|
| 30A | F. | 5 | 240 | 12 | – | |
| 30B | F. | 5 | 240 | 10 | – | |
| 30C | F. | 5 | 320 | 26 | + | 0·35 |
| 31A | M. | 5 (2·5 × 2) | 345 | 30 | ++ | 1·0 |
| 32A | F. | 5 (2·5 × 2) | 280 | 100 | ++++ | 4·5 |
| 32B | F. | 5 (2·5 × 2) | 265 | 8 | – | |
| 1B | F. | 30 (15 × 2) | 197 | 11 | – | |
| 1C | F. | 30 (15 × 2) | 270 | 13 | – | |
| 1D | F. | 30 (15 × 2) | 215 | 13 | – | |
| 1E | F. | 30 (15 × 2) | 185 | 8 | – | |
| 2B | M. | 30 (15 × 2) | 215 | 11 | – | |
| 2C | M. | 30 (15 × 2) | 230 | 9 | + | 0·4 |
| 2D | M. | 30 (15 × 2) | 265 | 9 | + | 0·3 |
| 2E | M. | 30 (15 × 2) | 295 | 13 | – | |
| 3D | F. | 30 | 265 | 12 | – | |
| 3E | F. | 30 | 245 | 22 | + | 0·3 |
| 4B | M. | 30 | 325 | 12 | + | 0·3 |
| 4C | M. | 30 | 340 | 16 | ++ | 0·3 |
| 4D | M. | 30 | 360 | 16 | +++ | 0·6 |
| 4E | M. | 30 | 210 | 8 | + | 0·3 |
| 19A | M. | 100 | 305 | 11 | – | |
| 19B | M. | 100 | 365 | 15 | – | |
| 19C | M. | 100 | 275 | 7 | – | |
| 19D | M. | 100 | 410 | 15 | – | |
| 19E | M. | 100 | 255 | 6 | – | |
| 22D | F. | 100 (50 × 2) | 260 | 15 | – | |
| 22E | F. | 100 (50 × 2) | 220 | 8 | – | |

+, ++, +++ as in Table II.
++++ represents adenomatous replacement of one or both lobes.

droplets similar to those noted in the methylthiouracil treated rats. No pituitary adenomas were encountered.

100 $\mu C$:

There were seven survivors. All of their thyroids without exception were made up of small follicles about 35$\mu$ across. There were practically no follicles larger than $50\mu$ in diameter. A few were lined by an extremely flattened epithelium and filled with deeply eosinophilic colloid. The majority presented a small lumen containing watery colloid and were lined by a tall epithelium. Bizarre shaped syncytial " follicles " were present in all the glands (Fig. 10). The peripheral follicles in general appeared as active as the central ones (Fig. 11). There was a well-marked variation in nuclear size ; some were unduly large and abnormal in shape (Fig. 10). The parathyroids appeared normal. Many arterioles were thick walled and hyalinised. No adenomas were seen. The body weights (Table III) were normal, the thyroid weights were reduced to about half normal.

The pituitaries were abnormal. They contained alpha cells in fair number but the beta cells were swollen, partially hyalinized and depleted of normal granulations. They were rich, however, in coarse mucoid granules and droplets (Fig. 12). Two small beta cell adenomas were encountered in each of the glands of two male rats.

---

### EXPLANATION OF PLATES.

FIG. 1.—Thyroid of male control rat 18F showing central follicles rich in colloid and lined by a cuboidal epithelium. × 480.

FIG. 2.—Thyroid of male control rat 18F showing larger peripheral than central follicles and a central cluster of microadenomas. × 36.

FIG. 3.—Thyroid of male control rat 18F showing microfollicles within the microadenomas. × 360.

FIG. 4.—Thyroid of female methylthiouracil rat 13E showing small colloid poor follicles and hypertrophied cells. × 360.

FIG. 5.—Thyroid of female methylthiouracil rat 13E showing active peripheral as well as central follicles and 2 adenomas. × 29.

FIG. 6.—Pituitary of female methlythiouracil rat 40A showing enlarged pars anterior cells, coarse (vesiculate) granulations and hyalinized cells. Periodic acid Schiff. × 480.

FIG. 7.—Pituitary of male control rat 18E showing normal size of pars anterior cells and absence of vesiculates and of hyalinization. Periodic acid Schiff. × 480.

FIG. 8.—Thyroid of female 5 $\mu$C $I^{131}$ rat 32A showing edge of large adenoma. × 52.

FIG. 9.—Thyroid of male 30 $\mu$C $I^{131}$ rat 4D showing moderately hypertrophied follicles and numerous adenomas. × 36.

FIG. 10.—Thyroid of male 100 $\mu$C $I^{131}$ rat 19B showing irregularly shaped small follicles poor in colloid, hypertrophied cells and bizarre nuclei. × 480.

FIG. 11.—Thyroid of male 100 $\mu$C $I^{131}$ rat 19B showing small active peripheral as well as central follicles. The gland is shrunken and contains an embedded parathyroid. × 36.

FIG. 12.—Pituitary of male 100 $\mu$C $I^{131}$ rat 19C showing coarse vesiculates and hyalinized cells. × 480.

FIG. 13.—Thyroid of male 5 $\mu$C $I^{131}$ methylthiouracil rat 37D showing hyperplasia and numerous adenomas. × 32.

FIG. 14.—Thyroid of female 30 $\mu$C $I^{131}$ methylthiouracil rat 5D showing malignant invasion and thrombosis of a pericapsular vein. × 144.

FIG. 15.—Thyroid of female 30 $\mu$C $I^{131}$ methylthiouracil rat 9D showing permeation of pericapsular veins by carcinoma. × 45.

FIG. 16.—Thyroid of female 30 $\mu$C $I^{131}$ methylthiouracil rat 6E showing carcinomatous breakthrough into the lumen of a large pericapsular vien. × 72.

FIG. 17.—Lung of male 30 $\mu$C $I^{131}$ methylthiouracil rat 10C showing permeation of a pulmonary artery by thyroid carcinoma and a large parenchymatous malignant deposit. × 50.

FIG. 18.—Thyroid of female 30 $\mu$C $I^{131}$ methylthiouracil rat 6D showing invasion of gland edge and capsule by scirrhous adenocarcinoma. × 90.

1
2
3
4
5
6

7

8

9

10

11

12

13

14

15

16

Doniach.

17

18

Doniach.

*5 μC $I^{131}$ and Methylthiouracil.*

There were 17 survivors. Their thyroids differed from those of the rats treated with methylthiouracil alone in the development of extremely numerous adenomas (Fig. 13), many of which were very large (Table IV). In addition,

TABLE IV.—*5 μC $I^{131}$ + Methylthiouracil.*

| Rat. | Sex. | Body weight (g.). | Thyroid weight (mg.). | Adenomas. | Diameter of largest adenoma (mm). |
|---|---|---|---|---|---|
| 33A | M. | 145 | 76 | ++++ | 2·5 |
| 33B | M. | 185 | 302 | ++++ | 4·0 |
| 33C | M. | 195 | 97 | +++ | 3·0 |
| 33D | M. | 175 | 93 | +++ | 2·5 |
| 33E | M. | 208 | 262 | ++++ | 6·0 |
| 35B | F. | 183 | 190 | ++++ | 4·0 |
| 35C | F. | 153 | 215 | ++++ | 5·0 |
| 35D | F. | 150 | 46 | +++ | 1·25 |
| 36A | M. | 136 | 84 | +++ | 2·0 |
| 36B | M. | 188 | 221 | ++++ | 4·0 |
| 36C | M. | 216 | 281 | +++ | 4·0 |
| 36D | M. | 202 | 129 | +++ | 1·0 |
| 36E | M. | 215 | 141 | +++ | 2·5 |
| 36F | M. | 178 | 119 | +++ | 0·5 |
| 37C | M. | 235 | 142 | +++ | 2·5 |
| 37D | M. | 148 | 227 | +++ | 5·0 |
| 37E | M. | 185 | 227 | ++++ | 4·0 |

The rats in Cages 35, 36 and 37 were given the iodine in 2 injections of 2·5 μC, the second injection at 7 months after a rest of 4 weeks from the methylthiouracil. The methylthiouracil was reinstituted 24 hours after the second injection of $I^{131}$.

+, ++, +++, ++++ as in Tables II and III.

only a few glands showed capsular fibrosis and the arterioles appeared normal. The follicles of the non-adenomatous areas presented a mixed picture. They were small, poor in colloid and lined by hypertrophic cells, some very similar to those seen in rats treated by methylthiouracil alone though not so hypertrophied. The majority, however, presented a closer affinity to those seen in the rats treated with 5 μC $I^{131}$ alone except that the cells were a little taller. Their nuclei varied in size and chromatism. The adenomas presented an exaggerated and bewildering variety of cell types and differentiation fundamentally similar to those produced by methylthiouracil alone. Some of the very large adenomas appeared to be derived from the confluence of more than one tumour. Colloid secretion was well marked in many tumours and a papillary arrangement was not rare. However, the major part of the bulk of even the large tumours was cellular rather than secretory in origin. Mitoses were not rare. Solid areas of undifferentiated spheroidal cells were seen within some tumours as with methylthiouracil alone. In Rat 35B tongues of adenoma tissue were seen penetrating the capsule at one point. However, in the absence of evidence of permeation of extra-capsular veins or obvious morphology of cancer, malignancy was not diagnosed. The body weights were similar to those of rats treated with methylthiouracil alone. But there was an even greater variation in the thyroid weights. The adenomas were significantly larger as well as more numerous. In 7 of the rats the largest adenoma exceeded the previous maximum diameter of 3·5 mm.

The pituitaries showed the same changes observed in the rats treated with methylthiouracil alone. No adenomas were encountered.

### *30 μC $I^{131}$ and Methylthiouracil.*

There were 20 survivors. Their thyroids showed a moderate capsular fibrosis and thickening of arteriolar walls, but much less marked than in the rats treated with methylthiouracil alone. Most of the follicles were small, bizarre shaped and lined by plump irregularly shaped cells showing irregularly arranged nuclei of varying sizes, shapes and chromatism. Their lumens, which were often slit shaped, contained a little colloid. The perifollicular capillaries were prominent, distended and indented the follicular cells. The majority of the follicles appeared active but could be differentiated from those seen in rats treated with methylthiouracil alone, as they were smaller, less regular and showed a tendency to a syncytial arrangement of their lining cells. Adenomas were present (Table V),

TABLE V.—*30 μC $I^{131}$ + Methylthiouracil.*

| Rat. | Sex. | Body weight (g.). | Thyroid weight (mg.). | Adenomas. | Diameter of largest adenoma (mm). |
|---|---|---|---|---|---|
| 5C | F. | 153 | 97 | +++ | 1·0 |
| 5D | F. | 197 | 320 | ++++ Ca | 8·0 |
| 6B | F. | 180 | 98 | +++ | 2·5 |
| 6C | F. | 113 | 80 | ++++ | 4·5 |
| 6D | F. | 144 | 88 | +++ Ca | 3·5 |
| 6E | F. | 170 | 91 | ++++ Ca | 4·0 |
| 7C | M. | 265 | 103 | +++ | 0·5 |
| 7D | M. | 245 | 221 | ++++ | 6·5 |
| 7E | M. | 240 | 147 | ++++ | 4·0 |
| 8B | M. | 220 | 136 | +++ | 1·25 |
| 8C | M. | 225 | 110 | +++ | 1·5 |
| 8D | M. | 210 | 82 | +++ | 0·5 |
| 8E | M. | 195 | 131 | +++ | 1·5 |
| 9D | F. | 161 | 350 | ++++ Ca | 5·5 |
| 9E | F. | 151 | 92 | +++ | 2·0 |
| 10C | M. | 200 | 540 | ++++ Ca | 8·0 |
| 10D | M. | 210 | 250 | ++++ | 6·5 |
| 10F | M. | 170 | 147 | +++ | 3·0 |
| 11D | M. | 230 | 153 | ++++ | 2·0 |
| 11E | M. | 195 | 175 | ++++ | 2·0 |

+, ++, +++, ++++ as in Tables II, III and IV. Ca represents malignancy.

The rats in Cages 5, 6 and 7 were given 2 injections of 15 μC $I^{131}$. The second injection was given 7 months after a 4 weeks' rest from the methythilouracil which was reinstituted 24 hours later.

similar in number, varied morphology and size to those found in the rats treated with 5μC $I^{131}$ plus methylthiouracil. In addition, there was evidence of thyroid malignancy in 5 animals. Rats 5D (Fig. 14), 9D (Fig. 15) and 6E (Fig. 16) showed permeation of large extracapsular thyroidal veins by tumour masses of spheroidal-celled growth showing some attempt at tubule formation. The lungs of Rat 10C (Fig. 17) contained numerous deposits of growth identical in morphology to the massive tumour in its thyroid. Rat 6D (Fig. 18) showed transformation of the pole of one lobe into a scirrhous poorly differentiated adenocarcinoma. It was not possible to tell from the morphology alone of any thyroid tumour whether it would prove invasive or not. It is interesting to note that all 3 of the thyroids which weighed more than 300 mg. showed evidence of malignancy. With one exception, the massive glands owed their bulk to the growth of one very large adenoma rather than to the presence of multiple tumours. The exception, 5D showed replacement and gross enlargement of one lobe by two large adenomas.

The cancers were more common in the females, 4 in 8 rats, than in the males, 1 in 12. But the numbers involved are too small for serious consideration of their significance. The body weights were similar to those of rats treated with methylthiouracil alone. The thyroid weights showed an even greater variation than that observed in the other groups.

The pituitaries were similar to those of the rats treated with methylthiouracil alone. They were free of adenomas.

### 100 $\mu C$ $I^{131}$ *and Methylthiouracil.*

There were 7 survivors. Their thyroids were made up entirely of small irregularly shaped follicles, some of which contained a little colloid. The lining cells were moderatley hypertrophic, and showed similar nuclear changes to those seen in the glands of rats treated with 100 $\mu$C $I^{131}$ alone. The perifollicular caplliaries were engorged and tended to indent the follicluar cells so as to contribute to alteration of their shapes. Arteriolar hyalinization was seen in 2 glands. The parathyroids all appeared normal. Adenomas mostly rather small, were seen in 4 of the glands (Table VI). They were of the same mixed nature seen in the other groups and showed occasional mitoses. The rats' weights were similar to those treated by methylthiouracil alone, but the thyroid weights were very considerably reduced to an average size comparable with that of the rats treated with 100 $\mu$C $I^{131}$ alone.

TABLE VI.—100 $\mu C$ $I^{131}$ + *Methylthiouracil.*

| Rat. | Sex. | Body weight (g.). | Thyroid weight (mg.). | Adenomas. | Diameter of largest adenoma (mm). |
|---|---|---|---|---|---|
| 24A | F. | 104 | 6 | — | |
| 24B | F. | 147 | 9 | + | 0·2 |
| 24C | F. | 163 | 17 | + | 0·3 |
| 24D | F. | 113 | 5 | + | 0·3 |
| 24E | F. | 130 | 11 | — | |
| 27D | F. | 210 | 33 | +++ | 1·0 |
| 27E | F. | 133 | 13 | — | |

+, +++ as in Tables II, III, IV and V.
The rats in Cage 27 recieved 2 injections of 50 $\mu$C $I^{131}$, the second 7 months after a 4 weeks' rest from methylthiouracil. The drug was reinstituted 24 hours after the second injection of $I^{131}$.

The pituitaries were similar to those of the rats treated with methylthiouracil alone. They showed no adenomas.

### *Measurement of Thyroid Uptake of Radioactive Iodine.*

Eight male rats aged 14 weeks were each injected intraperitoneally with 9·2 $\mu$C of $I^{131}$ carrier free in 1 ml. of water. In addition, 1 ml. was injected with the same syringe into a glass vial containing a small pledgelet of cotton-wool. Fifteen hours later *in vivo* measurements were made of the radioactivity of the rats' thyroids and of the 1 ml. standard solution which was placed against the counter in the position normally taken by the rats' necks. The average count rate obtained of the rats' thyroids was 2119 $\pm$ S.D. of 456. The count rate of the standard was 10,700, giving an average thyroid uptake at the end of 15 hours of 21 per cent. Nine hours later 4 of the rats were put on to a 0·1 per cent suspension of methylthiouracil in their drinking tap water. Daily *in vivo* measurements were then made of all the rats' thyroids for the ensuing 4 days. The results are summarised in Fig. 19. The upper curve is the calculated loss of radioactivity

FIG. 19.—Chart showing the average *in vivo* count-rates of radioactivity of the thyroid glands of 8 rats following an intraperitoneal injection of 9·2 $\mu$C $I^{131}$. After 24 hours 4 of the rats (lowermost curve) were put on to methylthiouracil in their drinking water. The findings in the remaining 4 are charted in the middle curve ; the calculated loss of radio-activity due solely to the radioactive decay of $I^{131}$ is charted in the uppermost curve. Count-rate of standard at 15 hours was 10,700.

due to the normal radioactive decay of $I^{131}$. The middle curve shows that after about 30 hours there is an increasing loss of radioactivity from the thyroid, due to release of organically bound iodine. The lower curve confirms the well established increased rate of loss of bound iodine from the thyroids of animals treated with antithyroid drugs.

### DISCUSSION.

### *Effects of internal irradiation by $I^{131}$ on thyroid function.*

Among the known functions of the thyroid cell are ability to concentrate iodine, to synthesize thyroxine, to hold the hormone in storage, to release the hormone from the follicle, to respond to pituitary stimulation by hypertrophy and by hyperplasia. The differential effects on these functions of varying doses of $I^{131}$ have been recently reported in 3 publications.

Skanse (1948) studied the effects of 1, 10 and 50 $\mu$C $I^{131}$ on young cockerels primed with a short course of thyrotrophic hormone. He found that the $I^{131}$ was equally well concentrated by the thyroids at all doses, 12·5 to 13·5 per cent at 24 hours ; but that the chicks injected with 50 $\mu$C $I^{131}$ showed a lower percentage concentration of $I^{131}$ in the thyroid than in the other groups after 96 hours, which he considered due to a more rapid release of hormone as a result of irradiation damage. A definite inhibition of normal thyroid growth was produced by 10 $\mu$C and 50 $\mu$C $I^{131}$ after 16 days but not of body growth. All the thyroids showed a growth response to thiouracil, less marked in the chicks given the higher dosages of $I^{131}$. But this response to thiouracil was much more inhibited after 38 days than after 26 days. Skanse (1948) remarks that though the major part of the radiation was received in the first few days, it took a much longer time to produce the full biological effect on gland function. The calculated dosages of total radiation to the thyroid glands were 1700 rep in the 1 $\mu$C group, 13,000 rep in the 10 $\mu$C group and 60,000 rep in the 50 $\mu$C group.

Feller, Chaikoff, Taurog and Jones (1949) studied the effects on adult male rats of single intraperitoneal doses of 24, 300 and 875 $\mu$C of $I^{131}$. The average uptake of $I^{131}$ into the thyroids was 40 per cent between 23 and 48 hours after injection and was about equal in all the animals. Those treated with the two higher doses showed an abnormally rapid depletion of the iodine that had been trapped during the first 24 to 48 hours. Chemical analyses showed an increasing depletion of thyroid $I^{127}$ from 2 to 3 days onwards after the injection of the 300 and 875 $\mu$C $I^{131}$, whereas there was no loss after 6 days in animals given 24 $\mu$C. The values of plasma protein bound iodine were not changed in the 24 $\mu$C group but they fell steadily in the rats injected with 300 $\mu$C from 3·6 $\mu$g. initially to 2·4 $\mu$g. per 100 ml. at 8 days. After an initial fall the plasma protein bound iodine showed a subsequent small temporary rise in the rats injected with 875 $\mu$C. This was associated with a fall in thyroid organically bound iodine presumed due to dissolution of thyroid protein induced by the irradiation. Thence followed a continuous severe fall to 1·5 $\mu$g. per 100 ml. plasma. The effect of the irradiation on the iodine concentrating capacity of the glands was tested at 3 and 10 days by a subsequent injection of $I^{131}$. No subsequent inhibition was found after an initial dose of 30 $\mu$C. But 300 $\mu$C deprived the thyriods of most of their capacity to concentrate iodine when tested after 3 and 10 days. Feller *et al.* (1949) calculated that the total irradiation doses to the thyroids were 19,000 rep at the centre and 8000 rep at the surface of the glands in the 24 $\mu$C group at 6 days ; 180,000 at the centre and 73,000 at the surface at 8 days in the 300 $\mu$C group ; 290,000 at the centre and 120,000 at the surface of the thyroids at 8 days in the 875 $\mu$C group.

Maloof, Dobyns and Vickery (1952) carried out extensive investigations on 500 young rats fed a low iodine diet for 14 days preceding the injections of 1, 5, 20, 50, 100 and 300 $\mu$C $I^{131}$, and which were subsequently maintained on a high iodine diet. As a result of the low iodine diet, thyroid uptake of $I^{131}$ was high, 47 to 62 per cent, and proved to be relatively uniform in all the groups. The rate of loss of $I^{131}$ from the glands increased significantly however, with the amount of radioactivity administered and was extremely rapid in the 300 $\mu$C group. Rats tested 48 days after the initial injection of $I^{131}$ showed a similar thyroid uptake of a tracer dose of $I^{131}$ in all groups while on a high iodine diet. They responded less well to a subsequent 11-day course of a low iodine diet. The thyriod uptake in the 1 $\mu$C reached 53 per cent 48 hours after receiving a tracer dose of $I^{131}$, but was significantly less in all the other groups. Response to subsequent thiouracil was similarly impaired when tested. The thyroids of animals which had received 50 and 100 $\mu$C $I^{131}$ demonstrated no capacity to increase in weight on a 30-day course of thiouracil instituted 64 days after the initial dose of radioactive iodine. Even 5 $\mu$C initial $I^{131}$ impaired the capacity of the thyroid to increase in size ; the glands enlarged from about 5·5 mg/100 g. body weight to 10·3 mg. as compared with 14·99 mg. in the animals given an initial dose of 1 $\mu$C $I^{131}$. Measurements of body weight showed no disturbance in animals which had received up to 20 $\mu$C, a slight impairment in the 50 and 100 $\mu$C groups and a marked disturbance in the 300 $\mu$C group. The calculated maximum total irradiation doses delivered to the thyroid in rep were respectively 1800 in the 1 $\mu$C, 5800 in the 5 $\mu$C, 30,000 in the 20 $\mu$C, 80,000 in the 50 $\mu$C, 197,000 in the 100 $\mu$C and 288,000 in the 300 $\mu$C groups.

*Effects of internal irradiation by $I^{131}$ on thyroid histology.*

Findlay and Leblond (1948) studied the thyroid histology of two rats killed 6 days after a single injection of $I^{131}$ calculated to have given a total thyroid irradiation of about 20,000 rep. The rats had previously been maintained on a low iodine diet. Most of the follicles, especially the central ones had lost their colloid, and were replaced by irregularly arranged solid groups of epithelial cells, some of which showed nuclear and cytoplasmic degeneration. Interstitial oedema was pronounced, fibrosis and lymphocytic infiltration slight. Many follicles in the periphery and isthmus were normal Autoradiographs showed evidence of accumulation of iodine only in the surviving colloid containing follicles of the periphery. Goldberg, Chaikoff, Lindsay and Feller (1950) studied the progressive changes in the thyroids of adult rats given varied doses of $I^{131}$ and killed after intervals of 1 day, 2 days, 3 days, 1 week, 2 weeks, 3 weeks, 1 month, 6 months and 8 months. 875 $\mu$C (330,000 rep to gland centre at 72 hours) produced a total destruction of the thyroids evident within 48 hours, associated with an intense fibrinous oedema and acute inflammatory changes, followed by fibroblastic proliferation and arterial thrombosis. By 3 weeks there was a total collagenous permeation of the original gland structure, which by 6 months was replaced by a band of hyalinised collagen. 525 $\mu$C produced essentially similar changes. There were, however, at 5 months a few surviving thyroid follicles at the poles, lined by bizarre granular cells thought to resemble the so-called Hürthle cells of the human gland. At 8 months surviving thyroid cells appeared more numerous and formed occasional colloid containing peripheral follicles. 300 $\mu$C (110,000 rep at 72 hours) produced extensive swelling and desquamation of thyroid cells within 24 hours, followed during the next 2 days by further degeneration and by interstitial inflammation which appeared resolved by 8 days. At 4 months the glands were lobular and made up of mixed disturbed and normal follicles. At 8 months the glands appeared essentially normal, though the follicle cell nuclei were hyperchromatic and interstitial tissue was increased. No evidence of damage was seen in rats given 18 $\mu$C (12,500 rep at 9 days). Gorbman (1950) summarized his own previous studies on radiotoxic doses of $I^{131}$ to mice. He found a minimum thyroid-lethal dose to be 4 $\mu$C per mg. thyroid and that it required 2 to 3 weeks to destroy all thyroid tissue. Larger doses were effective more quickly; 20 $\mu$C/mg. produced total thyroid cell necrosis in 2 days. Following the administration of minimal thyroid-lethal doses, parenchymatous degenerative changes first appeared within a few days in the gland and were followed by an inflammatory picture. Surviving follicles disappeared within a few weeks. Intrathyroidal doses of 2 $\mu$C/mg. thyroid permitted survival of a few thyroid follicles made up of swollen bizarre cells which persisted over the ensuing $1\frac{1}{2}$ years. Autoradiographs showed a minimal uptake of $I^{131}$ localised to colloid.

Maloof *et al.* (1952) described the histology of the thyroids at varying time intervals after administration to rats of $I^{131}$ as a part of the study of functional changes quoted above. After 2 days parenchymal degeneration and interstitial inflammation were seen centrally in the 300 $\mu$C group ; no significant changes were seen in the other groups After 48 days the 300 $\mu$C group showed a virtual replacement by fibrous scar tissue. The other groups, including the rats given 1 $\mu$C, all showed hypertrophy of follicle cells and diminution of colloid. With the higher dosages occasional enlarged and bizarre follicle cell nuclei were observed. After 94 days and following a course of thiouracil many more large bizarre nuclei were seen and were then present in the 5 $\mu$C group. After 1 year the 1 $\mu$C group showed a normal histology. Impressive cellular hypertrophy persisted in the 5, 20, 50 and 100 $\mu$C groups. Follicles varied in size, colloid was sparse, larger cells showed an eosinophilic granular cytoplasm, atypical nuclei increased in frequency, proportional to the initial dose of radioactive iodine. Autoradiographs showed some uptake of $I^{131}$ in all rats, including the 100 $\mu$C group. After $1\frac{1}{2}$ years no further changes were seen except that one adenoma was found in one rat in the 5 $\mu$C group. Since the irradiated thyroids had a reduced functional capacity, the authors regarded the cellular hypertrophy to have arisen, in part, from the stimulus caused by a deficiency in thyroxine production. Since evidence of hyperplasia was lacking, Maloof *et al.* (1952) suggest that the mechanism of cellular division had been impaired by the irradiation. Stimulation by thiouracil administration accentuated the cellular changes produced by the radioiodine. The persistence of cellular hypertrophy was, therefore, thought to be due to a radiation-induced failure of any significant compensatory regeneration.

*Summary of literature on functional and histological changes induced by $I^{131}$.*

The concentration of iodine by the thyroid during the first 24 hours does not vary with the dose of carrier-free $I^{131}$. It is dependent on the iodine content of the rat's previous diet which conditions the thyroid's avidity for iodine. A dose of $I^{131}$, harmless to animals fed a high iodine diet, may prove destructive to the thyroids of rats previously fed a low iodine diet. The retention of radioactive iodine trapped in the thyroid is markedly affected by dosage. From 30,000 rep upwards there is an increase in rate of loss of organically bound iodine from the thyroid, associated with radiation damage to the follicles. In general, functional disturbances precede histological changes. From 5000 rep upwards the thyroid cells are able to respond to activating stimuli by hypertrophy, but hyperplasia proves abnormally limited. Bizarre thyroid cells are seen histologically up to $1\frac{1}{2}$ years and are accentuated by thiouracil treatment, increasing in number in thyroids submitted to 5800 rep upwards. The follicles of the periphery and isthmus are subjected to less radiation damage than the central ones. Even though irradiated thyroid cells are histologically abnormal, they appear by means of hypertrophy to synthesize enough thyroxine to maintain the animals' health and growth after dosages up to 30,000 rep.

*Calculation of dosage of radiation to the thyroid.*

Calculations of radiation dosage to the thyroid following the administration of radioactive iodine can at the best only be rough estimates because of the considerable variation in distribution of iodine and in uptake and output of $I^{131}$ from follicle to follicle in addition to biological variation between rats. From the point of view of carcinogenesis it was considered more important to attempt to assess the likely range of dosage to the thyroid in each group than the total maximal radiation. It was found that the 30 $\mu$C $I^{131}$ group of rats in the present experiment received a radiation dosage to the thyroid which lay within the same range aimed at in the treatment of humans with Graves' disease.

Feller *et al.* (1949) pointed out that since the maximum range of the beta particles emitted from $I^{131}$ is comparable to the dimensions of the rat's thyroid, a geometrical factor must be calculated to estimate the reduction in radiation dose at points near the surface of the gland as compared with the centre. By assuming the thyroid lobes to be spherical they calculated the factor to be 0·42 to 1 (surface to centre). By following the uptake of radioactive iodine into the thyroid and its sequential loss from the gland it is possible to estimate the total radiation dose. The conversion factor for rep was calculated by Evans (1947): 1 $\mu$C per mg. of tissue delivers 8·5 rep per minute. This method was used by Maloof *et al.* (1952) for doses from 1 to 300 $\mu$C and their findings have been applied directly to the present experiments since our decay curve was comparable with theirs. Corrections were made for the initial differing maximal thyroid concentrations of $I^{131}$ of 55 per cent in their rats and 21 per cent in ours. The calculation of the total thyroid radiation to the centres of the glands in the present experiment comes to 2700 rep for the 5 $\mu$C group, 16,200 for the 30 $\mu$C group and 60,000 for the 100 $\mu$C group. The dosages at the periphery are presumed to be 42 per cent of these figures (Feller *et al.*, 1949), i.e., 1130, 6800 and 25,200 rep respectively.

The rats put on to methylthiouracil 24 hours after the injection of radioactive iodine showed a more rapid iodine loss (Fig. 19) than controls and appear to have received about two-thirds of the maximal irradiation calculated above.

In previous calculations the wide variation in uptake from follicle to follicle, observed in all autoradiographs, has been neglected. The average range of beta rays emitted by $I^{131}$ is about 440 $\mu$ in tissues a length well above the normal diameter of the central follicles of the rat thyroid. The central follicles are thus exposed to a cross-fire which is likely to prevent the dosage from falling below an average level in the gland centre. But the cells which line central follicles, which are frequently observed in autoradiographs to take up more $I^{131}$ than the rest, are likely to be submitted to at least a 50 per cent higher dose. In contrast to this, cells lining peripheral follicles, some of which have a mean diameter of over 250 $\mu$ and which, in autoradiographs, show an $I^{131}$ uptake well below the others, are likely to be submitted to a dose of roughly half that calculated geometrically. Indirect evidence of this latter point is the finding, quoted above, of survival of peripheral follicle cells in the thyroids of rats injected with as much as 525 $\mu$C $I^{131}$ (Goldberg *et al.*, 1950). In view of these findings, 50 per cent was added to the radiation dosage calculated for the centres of the glands and 50 per cent subtracted from the dosage calculated for the gland peripheries. The final calculated ranges are as follows: 5 $\mu$C $I^{131}$—570 to 4050; 30 $\mu$C $I^{131}$—3400 to 24,300; 100 $\mu$C $I^{131}$—12,600 to 90,000 rep and one-third less in animals in which the radioactive iodine was followed by methylthiouracil, i.e., 380–2700, 2270—16,200 and 8400–60,000 respectively.

TABLE VII.—*Summary of All Experiments.*

| Treatment. | Number of rats. M. | F. | Mean body weight in g. + S. error of mean. | Mean thyroid weight in mg. + range of weights. | Thyroid adenomas : remarks. |
|---|---|---|---|---|---|
| Controls | 3 | 6 | 252 ± 23 | 20 (17 – 25) | Microadenomas in moderate number in 7 of the total 9. |
| Methylthiouracil | 7 | 13 | 178 ± 8·4 | 177 (87 – 285) | Adenomas in moderate number in 19 of the total 20. |
| 5 μC $I^{131}$ | 1 | 5 | 281 ± 17·5 | 31 (8 – 100) | Adenomas in moderate number in 3 of the total 6. |
| 30 μC $I^{131}$ | 8 | 6 | 258 ± 14·7 | 12 (8 – 22) | Adenomas in moderate number in 7 of the total 14. |
| 100 μC $I^{131}$ | 5 | 2 | 298 ± 25·3 | 11 (5 – 15) | No adenomas in any of the 7. |
| 5 μC $I^{131}$ + Methylthiouracil | 14 | 3 | 182 ± 7 | 167 (46 – 302) | Very numerous large adenomas in all 17. |
| 30 μC $I^{131}$ + Methylthiouracil | 12 | 8 | 194 ± 10 | 170 (82 – 540) | Very numerous large adenomas in all 20. 5 carcinomas of thyroid. |
| 100 μC $I^{131}$ + Methylthiouracil | – | 7 | 143 ± 13·4 | 13 (5 – 33) | Adenomas in moderate number in 4 of the total 7. |

The rats were killed 15 months after the administration of the radioactive iodine ; those treated with methylthiouracil were given the drug as a 0·1 per cent suspension in their drinking water for most of the 15 months.

*Comment on experimental findings.*

The finding of very numerous thyroid adenomas and of 5 carcinomas in the thyroids of the rats treated with 30 μC $I^{131}$ and methylthiouracil confirms the results reported previously (Doniach, 1950). Purves and Griesbach (1947) obtained thyroid adenocarcinomas in 7 out of 13 rats killed after 20 months' or more treatment with thiourea alone but no carcinomas in animals killed earlier. The radioactive iodine, in the present experiment appears therefore to have hastened the onset of malignancy. The animals were all killed after 15 months and the rats on methylthiouracil alone showed no carcinomas. 5 μC $I^{131}$ and methylthiouracil led to the production of a greatly increased incidence of adenomas, but no definite carcinomas. These findings confirm the additive action of radioactive iodine in carcinogenesis by antithyroid drugs, and show that the range of 2270 to 16,200 rep was more effective than 380 to 2700 rep. In contrast, the methylthiouracil given to rats treated with 100 μC $I^{131}$ produced less adenomas than methylthiouracil alone. This inhibition at a dosage level of 8400 to 60,000 rep confirms the findings of Maloof *et al.* (1952) of the greatly diminished capacity of heavily irradiated thyroid cells to undergo hyperplasia. Adenomas of a similar morphology to those present in the thyroids of rats treated with antithyroid drugs were found in the animals given 5 μC and 30 μC $I^{131}$ alone, the latter appearing more effective than the former. The dosages of 3400 to 24,300 and 570 to 4050 rep in these groups lie within the range demonstrated by Maloof *et al.* (1952) to interfere with thyroid function, and may have been, therefore, partly effective by leading through diminished throxine output to an increased thyrotrophic hormone production. 100 μC $I^{131}$ alone appears to have inhibited the capacity of the thyroid cells to undergo hyperplasia. The histological changes in the thyroids and pituitaries of this group are significant. There are two points of evidence that a certain amount of thyroxine was being synthetized. Firstly, the animals grew from an initial weight of 100 g., when the radioactive iodine was first administered, to the full weight of 250 to 350 g., at the end of the experiment. Secondly, the pituitaries showed a good complement of well granulated alpha cells. Purves and Griesbach (1946) demonstrated that the maintenance of the alpha granulations is dependent upon a supply of thyroxine ; the pituitary alpha cells disappear after thyroidectomy and are restored by the daily administration of one-fifth or more of the normal thyroxine requirements. Nevertheless, the beta cells showed the typical changes of a raised level of thyrotrophic hormone secretion described by Griesbach and Purves (1945). This suggests that the irradiated thyroid glands were secreting at least one-fifth but less than the normal thyroxine requirements. The diminished capacity of the irradiated glands to regenerate prevented a return to quantitatively normal thyroxine synthesis, thus perpetuating an increased thyrotrophic hormone output and accounting for the hypertrophy of the thyroid cells. Another possibility to be considered is that irradiated thyroid cells differ from normal in requiring a higher level of thyrotrophic hormone stimulation in order to secrete a normal quantity of thyroxine. Similar changes in the thyroids and pituitaries were observed to a lesser extent in the 30 $\mu$C $I^{131}$ treated rats and to a slight extent in the thyroids only of the 5 $\mu$C $I^{131}$ group.

The bizarre thyroid cell nuclei observed by Maloof *et al.* (1952) in $I^{131}$ treated rats were seen in the present experiment, and were also accentuated in rats treated by additional thiouracil. One cannot say whether the neoplastic cells arise from these or from their apparently normal neighbours. They do, however, constitute evidence of irreversible irradiation changes. We do not know the life span of thyroid cells, but it is almost inconceivable that they could survive and function without replacement for 15 months, a period of nearly one half the animal's life span. It is likely, therefore, that the cells of the irradiated glands divide. Neoplasms in general arise in tissues made up of actively dividing cells. They do not arise from irreversibly differentiated cells such as neurones or keratinized epidermal cells. The chances of the development of neoplastic thyroid cells following irradiation are likely to vary directly with the number of post irradiation mitoses which take place. This number will be increased in the thyroid by thyrotrophic hormone stimulation. The proportion of cells which become neoplastic is presumably related to the dose of irradiation. The absence of adenomas in the 100 $\mu$C $I^{131}$ treated group is considered to be due to a lower incidence of dividing cells following irradiation than that which followed in the 30 and 5 $\mu$C groups. Adenomas might have been found after a further 6 months since they were present, albeit in small number, in the 100 $\mu$C treated rats submitted to additional thyrotrophic hormone stimulation by a prolonged course of methylthiouracil. We know from Goldberg and Chaikoff's (1952) findings that cancers may arise in rats $1\frac{1}{2}$ to 2 years after 300 $\mu$C of $I^{131}$.

Griesbach, Kennedy and Purves (1945) and Purves and Griesbach (1947) found involution of thiourea-induced benign and malignant thyroid tumours in rats when the thyrotrophic hormone level was lowered by the later addition of thyroxine to the treatment. Goitrogen induced tumours were transplantable only into animals kept in a state of thyroxine deficiency (Bielschowsky *et al.*, 1949) and Purves *et al.* (1951) concluded that as the tumours were capable of progressive growth, invasion of surrounding tissues and the production of distant metastases, they were malignant but were not " autonomous " since they were dependent on a high level of thyrotrophic hormone for their continued growth. Purves *et al.* (1951) have since studied the behaviour of a rat thyroid adenoma on serial transplantation. The original tumour appeared during long term thiouracil administration. The transplants were made into thyroxine deficient rats and underwent changes with the eventual production of three histological types. One of these was a malignant anaplastic carcinoma transplantable into hosts without thyroxine deficiency. It was not dependent on a high level of thyrotrophic hormone, did not concentrate iodine, grew rapidly, and killed its hosts within a few weeks.

The main difference between the action of antithyroid drugs alone and combined with the carcinogen acetamidofluorene is that with the latter treatment thyroid tumours appear considerably earlier and in greater number. Bielschowsky (1949) found also that acetamidofluorene produced thyroid adenomas in the absence of a chemical goitrogen in the regenerating thyroids of subtotally thyroidectomised rats. Purves *et al.* (1951) have concluded that neoplastic cells arise spontaneously in the normal rat thyroid and that the carcinogen acetamidofluorene seems to act by speeding up their formation, their further development and growth, however, being dependent upon a high thyrotrophic hormone level. This has been produced experimentally by the prolonged administeration of antithyroid drugs, or by prolonged iodine deficiency (Wegelin, 1927). The results of the previous (Doniach, 1950) and the present experiments show that the action of radioactive iodine is comparable with that of the carcinogen acetamidofluorene. In addition, radioactive iodine on its own leads by partial inhibition of thyroxine synthesis to a prolonged rise in thyrotrophic hormone level. The tumours produced by the radioactive iodine alone were benign, and in combination with methylthiouracil were of low grade malignancy. From the description of Goldberg and Chaikoff (1952), the experimental thyroid carcinomas which resulted from an extremely intense irradiation with radioactive iodine appeared to be of a high grade malignancy. The possibility that a carcinoma of low grade malignancy might become anaplastic must be considered after the findings in the transplantation experiment of Purves *et al.* (1951); the probability however is unknown.

*Dangers of carcinogenesis to humans treated with $I^{131}$.*

After an exposure to 1800 rep from $I^{131}$, Maloof *et al.* (1952) found a normal thyroid histology in rats $1\frac{1}{2}$ years later. But following 5800 rep and upwards, hypertrophied thyroid cells and increasing numbers with abnormal nuclei were seen, accentuated by a short course of methylthiouracil. These cells persisted up to $1\frac{1}{2}$ years. Tracer doses of $I^{131}$ in clinical medicine fall into the first category, those therapy doses which lower thyroid function below normal into the second. From the experimental findings in rats it would appear that 9000 rep to the thyroid in Graves' disease must act chiefly by functional damage leading to diminished thyroxine production rather than by actual total destruction of the gland. This is borne out by the brief descriptions of Chapman and Evans (1949), Williams *et al.* (1949) and Shapiro (1950), who noted no histological evidence of gross destruction in the thyroids of thyrotoxic patients after treatment with radioactive iodine. The hyperplastic thyroid of the thiouracil treated rat is not strictly comparable with the hyperplastic thyroid of thyrotoxicosis. There is certainly as yet no clear-cut evidence that the latter is associated with a high blood level of thyrotrophic hormone. However, a dose of radioactive iodine sufficient to cure Graves' disease might interfere sufficiently with thyroxine synthesis as to lead to a raised production of thyrotrophic hormone. The conditions may thus be fulfilled for tumour production, i.e., an initial radiation damage followed by prolonged thyrotrophic hormone stimulation. One would expect the latent period in humans to be many years, and it is possible that in time thyroxine production and thyrotrophic hormone levels might return to normal and the chances of tumour production be much diminished. This could be ensured by deliberate thyroxine medication. On the other hand, the treatment of a relapse, after radiation therapy, by antithyroid drugs might prove an ideal stimulus for tumour development. On present data therefore the carcinogenic danger of radioactive iodine appears to be the initiation of irreversible radiation damage to the thyroid. This renders a greater number of cells liable to tumour formation when later stimulated by thyrotrophic hormone than in the non-radiated gland. A proportion of the resultant tumours are likely to be carcinomas, probably of a low grade malignancy. The dosage lies within the known carcinogenic range. Anaplastic carcinomas may develop, especially when very destructive doses of $I^{131}$ are used.

In view of the experimental findings in rats (of various strains and in different countries), it is probable that the present methods of clinical $I^{131}$ therapy in thyrotoxicosis may eventually prove carcinogenic. We shall have to follow treated patients for 15 to 25 years in order to verify this danger and find out what proportion of them develop thyroid carcinoma. Meanwhile, four precautions are suggested. First, as prescribed in many centres in this country (Pochin, 1952 ; Wayne, 1952 ; Fraser and Abbatt, 1952, personal communication), thyrotoxic patients under the age of 45 should only be treated with radioiodine when other methods of treatment are contra-indicated or when the expectation of life is less than 20 years. Secondly, the minimal dose of $I^{131}$ to produce remission should be administered. Thirdly, thyroxine medication should be instituted and maintained after the thyrotoxic symptoms are relieved. Fourthly, antithyroid drugs are strongly contra-indicated at any time after radioiodine therapy. How long thyroxine medication should be maintained could be assessed by blood or urine estimations of thyrotrophic hormone levels after a trial period of a few weeks' cessation from the medication.

The carcinogenic danger to humans of $I^{131}$ therapy in Graves' disease is by no means proven by the above findings in rats since we do not know the comparable susceptibility to neoplasia of the thyroid glands of the two species, since we cannot directly compare dosages of irradiation, and we cannot assume that the rat's methylthiouracil stimulated thyroid is similar to the human thyrotoxic gland. Nevertheless the findings must be taken seriously. The effective $I^{131}$ dose range in rats is comparable with the therapy dose range in humans in so far as it appears to have similar biological effects in reducing thyroid size without actual gland destruction and produces no very gross loss in thyroxine synthesis. The crux of the matter seems to lie in the degree of radiation damage to the thyroid cells. If one could show that the present dosage of $I^{131}$ used in Graves' disease renders the patients euthyroid without producing an irreversible interference with thyroxine synthesis, then the carcinogenic danger is likely to be very slight. If, however, future thyrotrophic hormone studies show a perpetuated increase above normal after treatment, the carcinogenic danger may be great.

### SUMMARY.

The carcinogenic potency on the thyroid of 5, 30 and 100 $\mu$C $I^{131}$ was tested on a total of 100 rats, alone and combined with a subsequent 15-month course of methylthiouracil. The radioactive iodine was found to increase the formation of thyroid adenomas as compared with controls in the 5 and 30 $\mu$C groups but not in the 100 $\mu$C group. Out of 20 rats treated with combined 30 $\mu$C $I^{131}$ and methylthiouracil 5 developed thyroid carcinomas. The radiation dosage range to the thyroid of 2270–16,200 rep in the latter experiment is likely to include the dosage aimed at, of about 9000 rep, in the treatment of Graves' disease. The carcinogenic danger of radioiodine therapy is regarded as due to the production of irreversible cells changes in the thyroid, which render them more liable than normal cells to tumour formation when stimulated to undergo hyperplasia. At the same time, a dosage strong enough to interfere with thyroxine synthesis by radiation damage to the thyroid leads indirectly to a prolonged increased output of thyrotrophic hormone from the pituitary. This constitutes a perpetual stimulus to hyperplasia of the thyroid cells. It is thought that the present dosage of $I^{131}$ used in the treatment of Graves' disease may eventually prove carcinogenic.

I am grateful to Drs. Russell Fraser, S. R. Pelc and Selwyn Taylor for their advice and criticism, to D. G. Arnott for the measurements of radioactivity, E. V. Willmot for the photomicrographs, and J. G. Griffin and L. J. Wright for the sections.

### REFERENCES.

Arnott, D. G., and Fossey, P.—(1952) *J. Physiol.*, **118**, No. 2, 18P.

Bielschowsky, F.—(1944) *Brit. J. exp. Path.*, **25**, 90.—(1945) *Ibid.*, **26**, 270.—(1949) *Brit. J. Cancer*, **3**, 547.

*Idem*, Griesbach, W. E., Hall, W. H., Kennedy, T. H., and Purves, H. D.—(1949) *Ibid.*, **3**, 541.

Catchpole, H. R.—(1949) *J. Endocrin.*, **6**, 218.

Chapman, E. M., and Evans, R. D.—(1949) *Med. Clin. N. Amer.*, **33**, 1211.

Doniach, I.—(1950) *Brit. J. Cancer*, **4**, 223.

Evans, R. D.—(1947) *Amer. J. Roentgenol.*, **58**, 754.

Feller, D. D., Chaikoff, I. L., Taurog, A., and Jones, H. B.—(1949) *Endocrinology*, **45**, 464.

Findlay, D., and Leblond, C. P.—(1948) *Amer. J. Roentgenol.*, **59**, 387.

Glücksmann, A.—(1951) *J. Path. Bact.*, **63**, 176.

Goldberg, R. C., and Chaikoff, I. L.—(1952) *Arch. Path.*, **53**, 22.

*Iidem*, Lindsay, S., and Feller, D. D.—(1950) *Endocrinolgy*, **46**, 72.

Gorbman, A.—(1950) *J. clin. Endocrin.*, **10**, 1177.

Griesbach, W. E.—(1941) *Brit. J. exp. Path.*, **22**, 245.

*Idem* and Purves, H. D.—(1943) *Ibid.*, **24**, 174.—(1945) *Ibid.*, **26**, 13.

*Idem*, Kennedy, T. H., and Purves, H. D.—(1945) *Ibid.*, **26**, 18.

Maloof, F., Dobyns, B. M., and Vickery, A. L.—(1952) *Endocrinology*, **50**, 612.

Pearse, A. G. E.—(1949) *J. Path. Bact.*, **61**, 195.—(1952) *Ibid.*, **64**, 791.

Pochin, E. E.—(1952) *Proc. Roy. Soc. Med.*, **45**, 335.

Purves, H. D., and Griesbach, W. E.—(1946) *Brit. J. exp. Path.*, **27**, 170.—(1947) *Ibid.*, **28**, 46.—(1951) *Endocrinolgy*, **49**, 244.

*Iidem*, and Kennedy, T. H.—(1951) *Brit. J. Cancer*, **5**, 301.

RAPER, J. R., HENSHAW, P. S., AND SNIDER, R. S.—(1951) 'Effects of External Beta Radiation,' edited by R. E. ZIRKLE, Mcgraw-Hill Book Company, Inc.
SEVERINGHAUS, A. E., SMELSER, G. K., AND CLARK, H. M.—(1934) *Proc. Soc. exp. Biol., N.Y.*, **31**, 1127.
SHAPIRO, M.—(1950) *Ann. west. med. Surg.*, **4**, 274.
SKANSE, B. N.—(1948) *J. clin. Endocrin.*, **8**, 707.
WAYNE, E. J.—(1952) *Proc. Roy. Soc. Med.*, **45**, 338.
WEGELIN, C.—(1927) *Schweiz. med. Wschr.*, **8**, 848.
WILLIAMS, R. H., TOWERY, B. T., JAFFE, H., ROGERS, W. F., AND TAGNON, R.—(1949) *Amer. J. Med.*, **7**, 702.
WOLFE, J. M., BRYAN, W. R., AND WRIGHT, A. W.—(1938) *Amer. J. Cancer*, **34**, 352.